# Isomerization of photo-Gb_3_ in phase-separated pore-spanning membranes alters Shiga toxin organization

**DOI:** 10.1016/j.bpj.2025.07.011

**Published:** 2025-07-12

**Authors:** Larissa Socrier, Somayeh Ahadi, Mark Skamrahl, Daniel B. Werz, Claudia Steinem

**Affiliations:** 1 Max-Planck-Institute for Dynamics and Self-Organization, Göttingen, Germany; 2 Technische Universität Braunschweig, Institute of Organic Chemistry, Braunschweig, Germany; 3 Georg-August-Universität, Institute of Organic and Biomolecular Chemistry, Göttingen, Germany; 4 Albert-Ludwigs-Universität Freiburg, Institute of Organic Chemistry, Freiburg, Germany

## Abstract

The lateral organization of Shiga toxin bound to a lipid membrane is significantly influenced by the fatty acid geometry of its receptor glycolipid globotriaosylceramide (Gb_3_), which is crucial for the protein’s internalization into the host cell. To control the lipid geometry, we used a photoisomerizable azobenzene derivative of Gb_3_ (photo-Gb_3_) that can be switched between a *trans*- and a *cis*-configuration under light. We reconstituted this photo-Gb_3_ into liquid-disordered (*l*_d_)/liquid-ordered (*l*_o_) phase-separated pore-spanning membranes (PSMs), creating freestanding bilayer parts (freestanding PSMs [f-PSMs]) composed of an *l*_o_ phase surrounded by an *l*_d_ phase on the pore rims. Upon UV irradiation, we observed small, mobile, and transient *l*_d_ domains in the f-PSMs, indicating a *trans*-to-*cis* isomerization of the photo-Gb_3_. The mean diffusion coefficient of the *l*_d_ domains was determined to be 0.014 ± 0.009 *μ*m^2^/s, from which we estimated a membrane surface viscosity of 12–21 × 10^−8^ Pa s m, indicative of an *l*_o_ phase. Before photoisomerization, Shiga toxin B (STxB) bound homogeneously to the *l*_o_ phase in the f-PSMs harboring the photo-Gb_3_. Upon *trans*-to-*cis* isomerization of the photo-Gb_3_, *l*_d_ lakes were again formed, and the homogeneous protein distribution turned into dynamic STxB density fluctuations, which are discussed in the context of the entry process of Shiga toxin into the host cell.

## Significance

The fatty acid structure of the receptor glycolipid globotriaosylceramide (Gb_3_) influences the organization of membrane-bound Shiga toxin, which can impact the entry of the toxin into the host cell. To control the fatty acid geometry of Gb_3_ in situ in a time-resolved manner, we employed a Gb_3_-azobenzene derivative. Reconstituted into phase-separated pore-spanning membranes, the light-induced *trans*-to-*cis* isomerization of the Gb_3_ derivative led to small and mobile liquid-disordered lipid domains and Shiga toxin density fluctuations.

## Introduction

Glycosphingolipids, including Gb_3_ (globotriaosylceramide), are a class of lipid molecules known for their significant biological and physiological functions. Found in the outer leaflet of the plasma membrane, they play central roles in various biological processes, such as cell-cell communication, signal transduction, and modulation of membrane biophysical properties. Gb_3_, in particular, gains prominence due to its function as a receptor for bacterial Shiga toxins, which are responsible for certain types of food poisoning and other disorders like hemolytic uremic syndrome, colitis, or bloody diarrhea (1,2). Shiga toxins are AB_5_ proteins (3) produced by *Shigella dysenteriae* and enterohemorrhagic *Escherichia coli* and are composed of an enzymatically active A subunit and five B subunits. The B subunits recognize Gb_3_ as the first step for the toxin uptake into the host cell. After this uptake, the toxin is translocated to the endoplasmic reticulum via a retrograde transport through the *trans-*Golgi network into the cytosol, where the A_1_ domain of the A subunit catalytically removes an adenosine moiety from the 60S ribosomal subunit (4,5).

To investigate the first step of Shiga toxin uptake, model membranes appear well suited (6,7,8). Using giant unilamellar vesicles (GUVs) and supported lipid bilayers (SLBs) composed of a fluid-phase lipid, sphingomyelin, and cholesterol doped with Gb_3_ (9), it was found that Shiga toxin binds solely to the liquid-ordered (*l*_o_) phase in these *l*_o_/liquid-disordered (*l*_d_) phase-separated bilayers. The lateral organization of the bound protein is significantly influenced by the Gb_3_ geometry. Shiga toxin B (STxB) high-density protein clusters and low-density protein areas were observed on SLBs containing Gb_3_ with a saturated C_24:0_ fatty acid (10,11). Such protein clusters, accumulating Gb_3_ molecules with a saturated fatty acid, might inhibit membrane bending (9) due to the overall rigid structure of the membrane (12,13). However, a C_24:1_ unsaturated fatty acid leads to protein clusters (14,15) that are separated by protein-free *l*_o_ and *l*_d_ domains. This protein arrangement is supposed to induce negative curvature, eventually leading to the observed Shiga toxin-induced invaginations (16).

One drawback of SLBs is their direct contact with the support, which reduces the mobility of the lipids and lipid domains (17,18), hampering a rapid reorganization of the membrane and protein arrangement. To overcome this drawback, we developed pore-spanning membranes (PSMs) (19). PSMs are composed of supported membrane regions (supported PSMs [s-PSMs]) and freestanding ones (freestanding PSMs [f-PSMs]), in which lipid domain mobility becomes readily visible on the timescale of milliseconds (20). This membrane system provides the required dynamics (21) to investigate how a change in Gb_3_ geometry influences the organization of membrane-bound Shiga toxin in a time-resolved manner.

To switch the Gb_3_ geometry, we recently attached an azobenzene group to its fatty acid (22). Photo-switchable lipids, such as azobenzene derivatives, allow the modulation of *l*_o_ and *l*_d_ domains simply by light (23), which is noninvasive. Upon UV irradiation, the *trans*-to-*cis* isomerization of the azobenzene group leads to a diminution of the area of *l*_o_ domains, indicating a reduced membrane order (24). Conversely, after *cis*-to-*trans* isomerization induced by blue light irradiation, the area of *l*_o_ domains increases as membrane order is partially restored. Domain rearrangement was found to be dependent on the photo-lipid concentration, the position of the azobenzene group in the hydrophobic core of the membrane (25), and the structure of the polar head of the photo-lipids (24).

Here, we used a synthetic Gb_3_ derivative harboring an azobenzene moiety to switch it between the *trans*- and *cis*-configuration, thus altering the geometry of the glycolipid. Employing confocal laser scanning microscopy (CLSM) experiments, we observed changes in membrane organization in the f-PSMs upon light irradiation. The altered Gb_3_ lipid geometry also significantly changed the Shiga toxin organization on the *l*_o_ phase of the membrane.

## Materials and methods

### Materials

**Figure 1:**
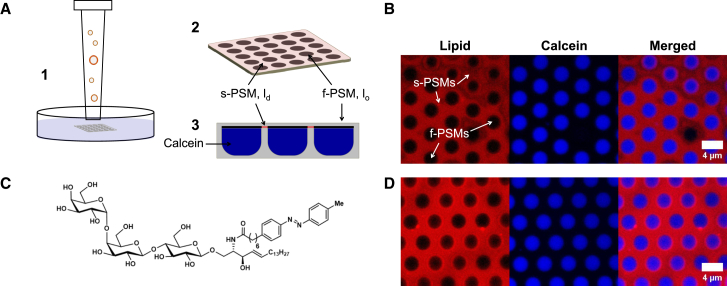
(*A*) Preparation of phase-separated pore-spanning membranes. 1) GUVs are sedimented on a SiO_x_-functionalized substrate in calcein-containing buffer at 65°C. 2) Samples were cooled down overnight, allowing the formation of *l*_o_ domains (*dark*) on the pores and *l*_d_ domains (*red*) on the pore rims. 3) Side view of the pores, in which calcein (*blue*) has been entrapped after the spreading of the GUVs, forming PSMs. (*B*) Fluorescence micrographs of phase-separated PSMs on a porous substrate with closed pores at the bottom (micro-cups). Lipid composition: DOPC/SM_18_/cholesterol/ATTO655-DOPE (39.5:39.5:20:1, *n/n*). Lipid: the *l*_d_ phase, labeled with ATTO655-DOPE (*red fluorescence*), is located on the s-PSMs, whereas f-PSMs are composed of the *l*_o_ phase from which the fluorescent dye is excluded. Calcein: the water-soluble dye calcein (*blue fluorescence*) is entrapped in the micro-cups. One pore is not covered by a membrane, which appears black. Merged: the lipid dye shows the s-PSMs, whereas the entrapped calcein reports on the pore coverage. In the bottom right corner, the pore is not covered with a membrane, and thus, calcein is not entrapped. (*C*) Chemical structure of photo-Gb_3_. (*D*) Fluorescence micrographs of phase-separated PSMs containing photo-Gb_3_. Lipid composition: DOPC/SM_18_/cholesterol/photo-Gb_3_/ATTO655-DOPE (37:20:22:20:1, *n/n*). A contrast correction factor (*γ*) of 0.5 was applied for better visualization.

1,2-dioleoyl-*sn*-glycero-3-phosphocholine (DOPC), *N*-stearoyl-D-erythro-sphingosylphosphoryl-choline (SM_18:0_), cholesterol, and BODIPY-cholesterol were purchased from Avanti Polar Lipids (Alabaster, Alabama). The photo-Gb_3_ (Fig. 1 *C*) was synthesized as described previously (22). ATTO655-DOPE was obtained from ATTO Technology (Siegen, Germany) and fluorescein-bis(methyliminodiacetic acid) (calcein) from Merck (Darmstadt, Germany). Silicon monoxide granules (SiO, 4–8 mm) were purchased from Merck. Indium tin oxide coverslips (25 × 75 mm^2^, thickness: 1.1 mm) were obtained from Präzisions Glas & Optik (Iserlohn, Germany). Porous silicon nitride micro-cup substrates with pore diameters of 3 *μ*m were obtained from Aquamarijn Membranes B.V. (Wageningen, the Netherlands). Phosphate-buffered saline (PBS) was prepared using 136 mM NaCl, 2.7 mM KCl, 8.1 mM Na_2_HPO_4_, and 1.5 mM KH_2_PO_4_. Calcein-PBS was prepared by dissolving the fluorophore in the buffer to a final concentration of 100 *μ*M. Buffers were adjusted to a pH of 7.4. All buffer reagents were purchased from VWR International (Darmstadt, Germany). Petri dishes (3 cm diameter) were purchased from Mattek (Ashland, Massachusetts).

### Preparation of GUVs

GUVs were prepared by electro-formation as described previously (26). Lipids were first dissolved in chloroform and the photo-Gb_3_ in chloroform/methanol (2:1, v/v). Mixtures containing DOPC/SM_18:0_/cholesterol/ATTO655-DOPE (39.5:39.5:20:1, *n/n*) or DOPC/SM_18:0_/cholesterol/photo-Gb_3_/ATTO655-DOPE (37:20:22:20:1, *n/n*) were prepared in a glass test tube and then spread on indium tin oxide coverslips to form dried lipid films (0.25 mg). The remaining solvent was removed by drying the coverslips under a vacuum at 65°C for 1 h. A homemade chamber was built using a silicon ring and filled with 1.7 mL of sucrose solution (298 mOsm/L). Electro-formation was performed for 3 h by applying an alternating sinusoidal current at a frequency of 12 Hz with an amplitude of 1.6 V (peak to peak). GUVs were collected in black microcentrifuge tubes and conserved for 5 days at room temperature. GUVs containing the chosen ternary lipid mixtures separate into coexisting *l*_o_/*l*_d_ phases as the temperature is lowered through the miscibility transition temperature *T*_mix_, which is around 40°C.

### SiO_x_ functionalization of porous substrates

The substrates were functionalized as described previously (27). Briefly, porous silicon nitride micro-cup substrates were rinsed with ethanol and ultrapure water and dried with a stream of N_2_. The substrates were placed in the center of a convex glass lens beaker, which was subsequently placed in a Bal-TecMed020 high vacuum coating system from Bal-Tec (Balzers, Liechtenstein). SiO was deposited on the substrates at a rate of 0.3 nm/s. Freshly coated substrates were placed in ultrapure water for 1 h at 55°C to form a hydrophilic SiO_x_ layer (x = 1–2).

### Preparation of PSMs

Preparation of the PSMs was carried out in the dark to protect the photo-Gb_3_ and at 65°C, well above the miscibility transition temperature *T*_mix_ of the lipid composition to ensure that the GUVs, spread on the porous support, are homogeneous and in the *l*_d_ phase. Freshly coated SiO_x_ substrates were glued in the center of petri dishes using non-conductive adhesive stickers (Plano, Wetzlar, Germany) and rinsed with ultrapure water. The petri dishes were filled with 3 mL of calcein-containing PBS. 150 *μ*L of the GUV suspension was sedimented on the substrate for 3 min with a 5 mL pipette tip filled with calcein-free PBS. The samples were then slowly cooled to room temperature overnight in the dark. During the cooling process, the PSMs separate into the *l*_d_ and *l*_o_ phases. Prior to imaging, samples were rinsed abundantly with calcein-free PBS buffer. For protein-binding experiments, STxB was labeled with the fluorophore Cy3 using a fast conjugation kit (ab188287, Abcam, Berlin, Germany). The labeled protein was added to the PSMs at a final concentration of 300 nM. After a gentle agitation for 1 h, the samples were rinsed with calcein-free PBS to remove unbound STxB.

### Light irradiation

A pE-4000 LED from CoolLed (Andover, Hampshire, United Kingdom) was used as a light source to irradiate the samples with UV-A (365 nm) and blue light (435–460 nm). The optical fiber was mounted in the collimator of the microscope so that the light could be directed to the samples via the optical path through the objective. The light power density (in W) applied to the samples was measured using a Field Max II-TO power meter from Coherent (Wilsonville, Oregon). Samples were irradiated for 15 s at a power density of 3 mW/cm^2^ with UV light and 150 mW/cm^2^ with blue light.

### Fluorescence microscopy imaging

CLSM experiments were carried out using an LSM 710 CLSM from Carl Zeiss (Jena, Germany) equipped with a 488 nm Ar laser (1% power), a 633 nm He-Ne laser (3% power), and a 561-10 DPSS laser (3% power) to excite calcein, ATTO655-DOPE, and the Cy3-labeled STxB, respectively. The pinhole was set to 1.5 airy units and the pixel dwell time to 1 *μ*s. Images and time series with 512 × 512 pixels^2^ (16-bit resolution) were recorded with a W Plan-Apochromat 63×/1.0 M27 objective. Image analysis was performed with FIJI (http://fiji.sc/Fiji (28)). Graphs were plotted with GraphPad Prism software 10 for Windows (San Diego, California).

### Image processing and domain diffusion analysis

To gather information about the diffusion of the observed *l*_d_ domains in the f-PSMs, the imaging data were analyzed using self-written Python-based scripts (29,30,31). Each f-PSM with a visible *l*_d_ domain was cropped from the movie with FIJI (28), and the consecutive images in which the *l*_d_ domain was visible throughout were subjected to the analysis. At first, a histogram-matching-based adjustment of the image intensities was performed to account for the bleaching of the fluorophore during image acquisition (32). Here, the intensities of all images in each time series were adjusted so that their intensity histogram matched the intensity histogram of the first image as closely as possible. Next, a Gaussian blur was applied to the images to reduce the impact of noise. A two-dimensional Gaussian kernel with a *σ* value of 70 nm was used for the convolution of all frames. Since the domains only appeared after UV irradiation, the frames before the UV irradiation were used for background correction using the same image-processing routine (cropping out the pore of interest, bleaching correction, and Gaussian blurring). The resulting frames were averaged and then subtracted from each image in which the domain was visible. This increased the contrast between the domains and the background and removed signals from the pore rims. After this preprocessing, a circular mask was applied to the images to exclude image features outside the pore as well as close to the pore rim. A thresholding-based binarization was performed by manually choosing an appropriate intensity threshold to track the domains using the coordinates of the domain centroid. The coordinates of the binarized domain were determined using OpenCV-based contour finding and image moments (33). Plotting was performed using Matplotlib and Trackpy (34,35). The mean-squared displacement (MSD) was calculated from the trajectories, and the diffusion coefficient *D* was extracted by fitting a linear function to the first four data points of each MSD as MSD(τ)=4Dτ with the lag time *τ*.

## Results and discussion

### Phase behavior of PSMs lacking photo-Gb_3_

PSMs were prepared by spreading GUVs composed of DOPC/SM_18_/cholesterol/ATTO655*-*DOPE onto SiO_x_-coated porous substrates. SiO_x_-coated porous substrates are advantageous over gold-covered ones because the intensity of the lipid fluorophores on the supported parts is much less quenched than on gold (20,36). GUV spreading was performed above the miscibility transition temperature of the lipid mixture and in the presence of a calcein-containing buffer (Figs. 1 *A* and S1). After slowly cooling the samples to room temperature overnight, which resulted in the phase separation of the lipid mixture, fluorescence micrographs were taken. The resulting fluorescence micrographs reveal PSMs comprising freestanding membrane parts (f-PSMs) and supported parts (s-PSMs). The s-PSMs are fluorescent, i.e., they harbor the fluid-phase marker ATTO655-DOPE (Fig. 1 *B*, lipid), indicative of the *l*_d_ phase. The fluorescence appears slightly inhomogeneous on the s-PSMs (Figs. 1 *B*, lipid, and S2, *A–C*). The lipid composition, in particular sphingomyelin, can influence the phase behavior of bilayers (37,38); however, the darker regions observed on the s-PSMs might result from an inhomogeneity of the SiO_x_ functionalization and a varying distance of the s-PSM from the support (39,40). In contrast, the f-PSMs appear dark, as the lipid label is excluded, indicating the *l*_o_ phase (39,40).

The observed phase separation of the lipid composition in PSMs agrees with our previous findings (39). In this study, we argued that the re-localization of the fluid-phase lipids onto the pore rims results from the adhesion strength of the lipids to the support (41) and the surface roughness of the SiO_x_ layer (RMS = 0.49 ± 0.03 nm) (40).

Sumitomo et al. (42) investigated phase-separated PSMs composed of DOPC/DPPC/cholesterol (30:40:30) on SiO_2_-coated porous substrates (RMS < 0.3 nm). However, they observed the fluid phase in the f-PSMs, which they partly explained with the overhang at the aperture of the substrate. With a smaller bending rigidity of the *l*_d_ phase than the *l*_o_ phase, the alignment of the membrane with the substrate topology would energetically be less costly (43). In addition, there is a difference in the overall surface roughness (SiO_2_ versus SiO_x_) and the lipid composition, with DOPC/DPPC/cholesterol (30:40:30) having an approximately 10°C lower miscibility transition temperature (44).

From the ATTO655-DOPE fluorescence micrographs alone (Fig. 1 *B*, lipid), it is difficult to evaluate whether f-PSMs were formed and whether the PSMs were impermeable. To select the areas on the surface where f-PSMs had been formed, the water-soluble dye calcein was entrapped into the cavities of the micro-cups. From fluorescence overlays of the lipid dye and calcein, the areas with f-PSMs were unambiguously identified (Fig. S1). The observed calcein fluorescence in the cavities clearly shows that f-PSMs with *l*_o_ phase, excluding the lipid dye, are present and entrap the water-soluble dye (Fig. 1 *B*, calcein, merged).

### Phase behavior and isomerization of Gb_3_-containing PSMs

**Figure 2:**
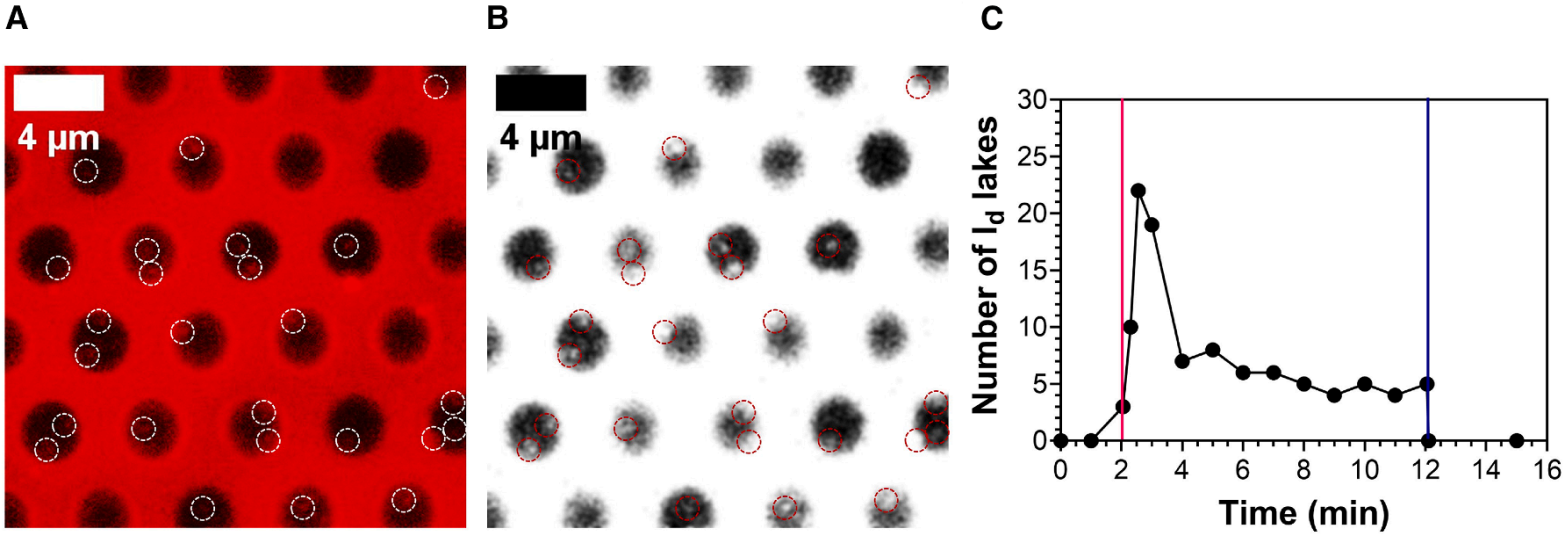
Isomerization of photo-Gb_3_ in protein-free PSMs. Lipid composition: DOPC/SM_18_/cholesterol/photo-Gb_3_/ATTO655-DOPE, 37:20:22:20:1, *n*/*n*. (*A*) Transient *l*_d_ lakes (*white dashed circles*) are visible in f-PSMs (*dark*) 30 s after *trans*-to-*cis* isomerization of photo-Gb_3_. (*B*) For better visualization of the *l*_d_ lakes (*red dashed circles*) and quantification, a grayscale image was generated with Gaussian blurring. (*C*) Quantification of *l*_d_ lakes during the illumination process (*N* = 27 f-PSMs). The number of *l*_d_ lakes increased immediately after UV irradiation (*magenta line*). The lakes persisted for a few seconds and partially vanished over time. Blue light irradiation (*blue line*) led to the complete disappearance of the remaining *l*_d_ lakes (Video S2).

Next, the influence of the photo-Gb_3_ on the stability and phase behavior of the PSMs upon isomerization was investigated. PSMs were prepared from GUVs containing 20 mol % of the photo-Gb_3_ (DOPC/SM_18:0_/cholesterol/photo-Gb_3_/ATTO655-DOPE) using the same protocol. Before irradiation, the fluorescence micrographs of the lipid fluorophore and the calcein were indistinguishable from those without the photo-Gb_3_ (Fig. 1 *D*). The PSMs were then exposed to UV-A and blue light irradiation, and the impact of isomerization on the membrane organization was investigated with CLSM. As a control, PSMs lacking the photo-Gb_3_ were irradiated (Fig. S2). In the absence of photo-Gb_3_, the appearance of the phase-separated PSMs remained unaffected, and no modification of membrane organization became visible (Fig. S2; Video S1). The calcein signal diminished significantly after blue light irradiation, which can be attributed to bleaching (Fig. S4, *A* and *B*), as this fluorophore absorbs in the blue region (45). In the presence of the photo-Gb_3_ and before irradiation, the PSMs appear the same as those lacking the azobenzene compound. The PSMs are phase separated, with the *l*_o_ phase mainly located on the f-PSMs (Fig. 1 *D*). Upon UV irradiation, small *l*_d_ domains became discernible in the f-PSMs (Fig. S3; Video S2). Such domains have been reported previously in SLBs and were termed *l*_d_ lakes (24,25). The analysis of the time-dependent calcein fluorescence in the cavities indicates that the f-PSMs did not become leaky upon UV irradiation (Fig. S4 *A* and *B*). Approximately 50% of the f-PSMs displayed at least one *l*_d_ lake 60 s after UV irradiation. We assume that the photo-Gb_3_ in the *trans-*configuration is preferentially localized in the f-PSMs, which are enriched in *l*_o_*-*phase lipids. Saturated Gb_3_ species were shown to be more miscible with *l*_o_-phase lipids (46), and photo-lipids in the *trans-*configuration behave more like saturated lipids (47). The *trans*-to-*cis* isomerization of the photo-Gb_3_ fluidizes small membrane areas leading to *l*_d_ lakes. Interestingly, a portion of the *l*_d_ lakes disappeared rapidly after UV irradiation. 5 and 10 min after UV illumination, *l*_d_ lakes were visible in approximately 30% and 20% of the f-PSMs, respectively (Fig. 2 *C*). The transient nature of the *l*_d_ lakes might be a result of the mobility of the lipids in the PSM system. The formed *l*_d_ lakes, including the *cis*-configured Gb_3_ molecules (48), equilibrate with the *l*_d_ phase on the s-PSMs and thus disappear over time (Fig. 2 *C*).

Video S1. Time-lapse of control PSMs after UV and blue light irradiation Frame rate: 20 fps.

Video S2. Time-lapse of photo-Gb3 PSMs after UV and blue light irradiation Frame rate: 20 fps.

**Figure 3:**
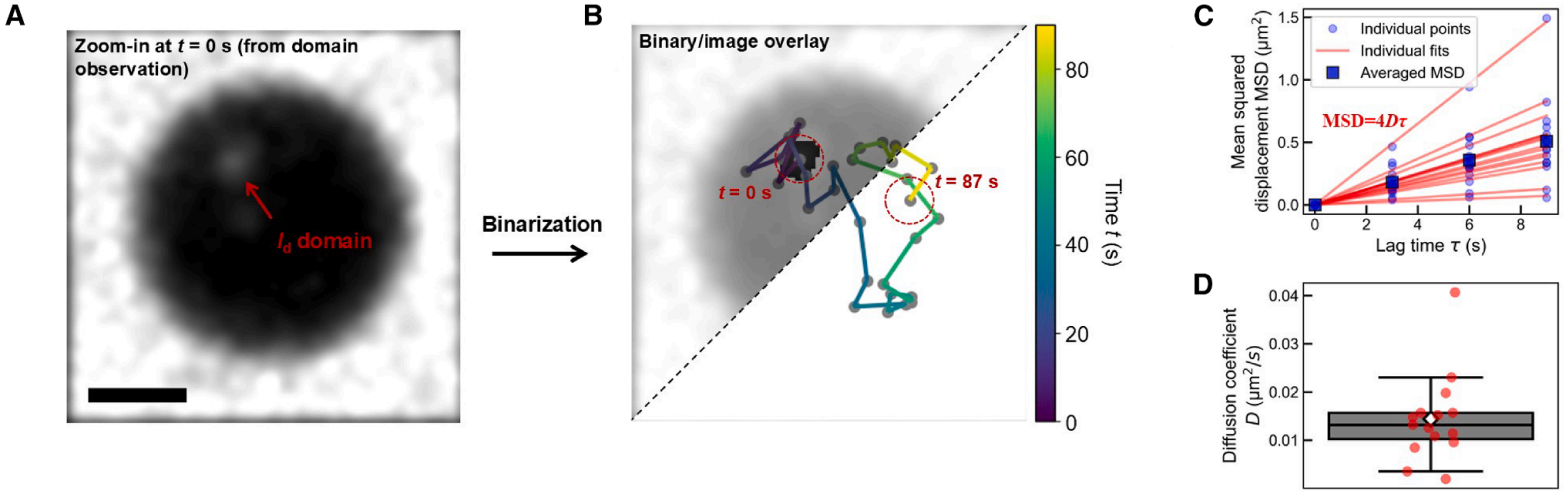
Diffusion analysis shows high mobility of the individual domains. (*A*) Greyscale image (after bleaching correction and Gaussian blurring) of an individual f-PSM with a mobile *l*_d_ lake (*red arrow*) right after it became visible (*t* = 0 s). Scale bar: 1 *μ*m. (*B*) Overlay of the image from (*A*) (*top left triangle*), the binarized *l*_d_ lake (*full image*; *black* corresponds to the recognized domain, whereas *white* was not recognized), and the corresponding trajectory. Red dashed circles indicate the location of the *l*_d_ lake at the start point (*t* = 0 s) and the end point (*t* = 87 s) of the trajectory. (*C*) Mean-squared displacement (MSD) curves of all analyzed domains (*n* = 15 single *l*_d_ lakes measured across 4 separate experiment days). Individual MSD data points are shown as blue circles. Red lines represent linear fits to the individual MSDs, yielding the diffusion coefficients shown in (*D*) as a boxplot. The box shows the quartiles of the *D* values, and the whiskers correspond to the 5^th^ and 95^th^ percentiles, whereas red circles indicate single measurements. The black line is the median. A mean diffusion coefficient of 0.014 ± 0.009 *μ*m^2^/s (*diamond*) was obtained.

Blue light irradiation led to the complete disappearance of the remaining *l*_d_ lakes, suggesting a successful *cis*-to-*trans* isomerization (Fig. 2 *C*; Video S2). On the s-PSMs, the membrane remains in the fluid phase during the entire illumination process (Fig. S3). This observation meets our expectations. s-PSMs are enriched in fluid-phase lipids, and because the *trans*-to-*cis* isomerization leads to fluidization (24,25), the phase does not change. In contrast to the *l*_d_ lakes in *l*_o_ domains in SLBs after *trans-*to-*cis* isomerization of photo-ceramides (25) and the uncaging of caged ceramides (49), the observed *l*_d_ lakes in f-PSMs were fully mobile (Fig. 3). To better visualize these individual *l*_d_ domains, we zoomed in on each corresponding f-PSM and applied image processing such as bleaching correction and Gaussian blurring (Fig. 3 *A*). To quantitatively analyze the *l*_d_-lake movement, we used an automated tracking algorithm. An exemplary track of an *l*_d_ lake is shown in Fig. 3 *B*. Even though, in this example, an *l*_d_ lake was visible for only 87 s, it moved almost across the whole f-PSM. To quantify this diffusion dynamics, we calculated the MSD and used a linear fit to determine the apparent diffusion coefficient of the *l*_d_ lake (Fig. 3 *C*).

From the MSD analysis, we calculated a mean diffusion coefficient of 0.014 ± 0.009 *μ*m^2^/s (Fig. 3, *C* and *D*). From the obtained diffusion coefficient, one can roughly estimate the membrane surface viscosity using the model of Hughes, Pailthorpe, and White (HPW) (50). Assuming free diffusion of the *l*_d_ lakes within the f-PSM, which is a valid assumption given the dimensions of the f-PSM with a radius of *r*_pore_ = 1.5 *μ*m and a radius of the *l*_d_ lakes, which is below the resolution limit of about 400 nm, one can neglect the confinement of the f-PSM as a first approximation (20) and apply the HPW model. They developed a hydrodynamical model applicable under an arbitrary tracer size and for arbitrary membrane viscosities (50). As the HPW model requires complicated numerical computations, Petrov et al. (51,52) proposed a semi-empirical formula describing the HPW model fairly well (Eqs. 1 and 2):

(1) $$D\left(\lambda \right)=\frac{k_{B}T}{4\pi \eta }\frac{\ln\left(2\lambda \right)-\gamma +\frac{4}{\pi }\lambda^{-1}-\frac{1}{2}\lambda^{-2}\;\ln\left(2\lambda \right)}{1-\left(\frac{1}{\pi }\right)\lambda^{-3}\;\ln\left(2\lambda \right)+c_{1}\lambda^{-b_{1}}/\left(1+c_{2}\lambda^{-b_{2}}\right)}\text{,}$$

with

(2) $$\lambda =\frac{\eta }{2\mu \;r_{\text{lake}\;}\;}\text{.}$$

*η* denotes the membrane surface viscosity, *μ* is the water viscosity, *k*_B_ is the Boltzmann constant, *T* is the temperature, and *γ* is Euler’s constant. The semi-empirically derived constants are as follows: *c*_1_ = 0.73761, *c*_2_ = 0.52119, *b*_1_ = 2.74819, and *b*_2_ = 0.51465. Using this model, one can estimate the membrane surface viscosity experienced by the *l*_d_ lakes. Assuming radii of the *l*_d_ lakes of *r*_lake_ = 10–400 nm, one obtains membrane surface viscosities of *η* = 21–12 × 10^−8^ Pa s m*,* which are significantly larger than the values obtained for inclusions in fluid-phase membranes (*η* = 0.2 × 10^−8^ Pa s m) (51). The large *η* supports the notion of small *l*_d_ lakes diffusing in an *l*_o_ phase (53) exhibiting a larger membrane surface viscosity.

### Isomerization of protein-enriched PSMs

**Figure 4:**
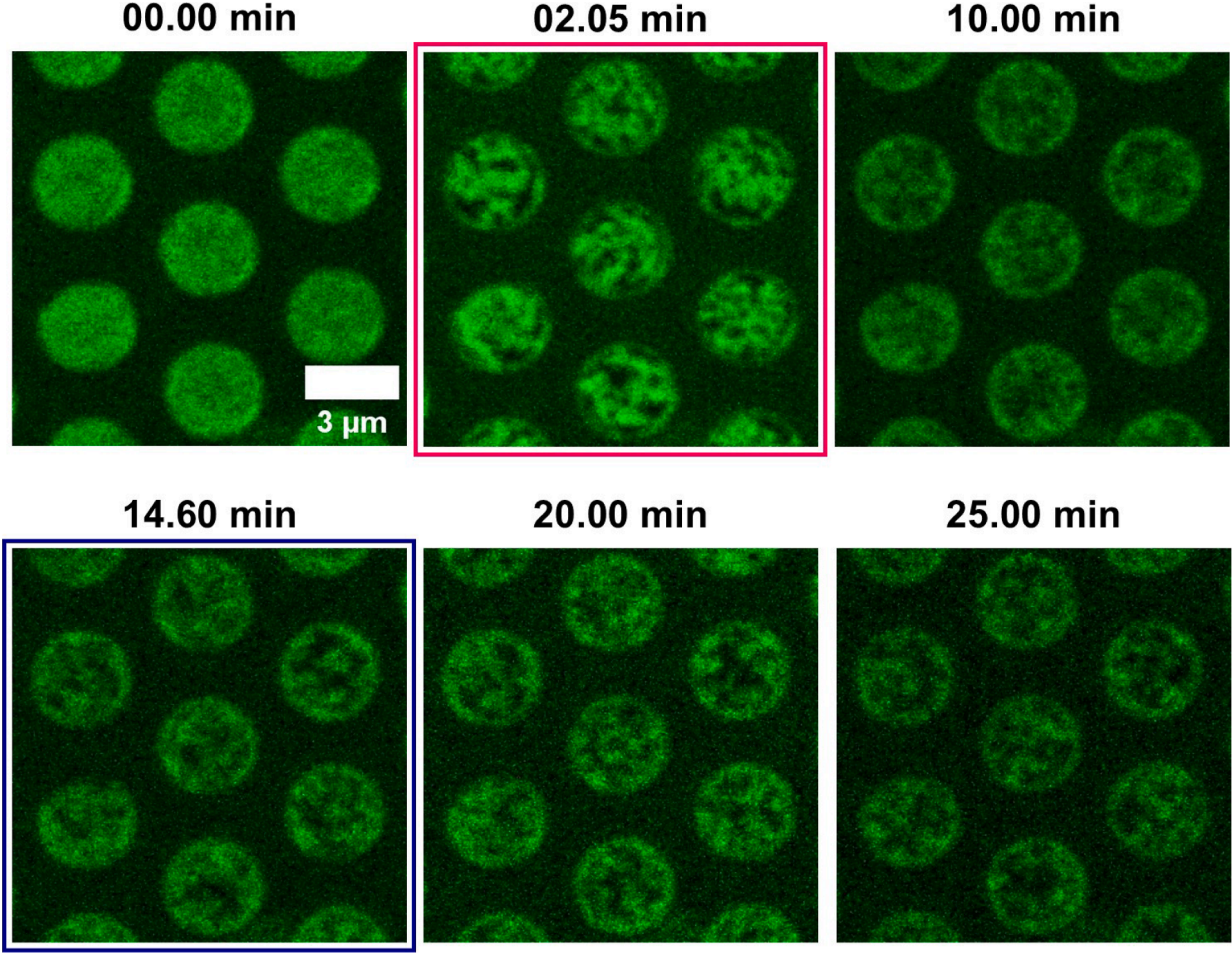
Light-induced formation of protein density fluctuations after the isomerization of photo-Gb_3_ in f-PSMs. Lipid composition: DOPC/SM_18_/cholesterol/photo-Gb_3_/ATTO655-DOPE, 37:20:22:20:1, *n/n*. *c*(STxB) = 300 nM. A time-lapse was recorded before and after light irradiation (Video S4). Before irradiation, STxB is homogeneously distributed on the surface of the f-PSMs. Protein density fluctuations appeared immediately after UV irradiation (*magenta*) and persisted after blue light irradiation (*blue*).

Based on the mobile f-PSMs with *l*_o_ phase, we next addressed how the Gb_3_ isomerization influences the lateral organization of membrane-bound STxB. PSMs containing 20 mol % of the photo-Gb_3_ (Fig. 1 *C*) were incubated with fluorescently labeled STxB (Fig. S5). The STxB signal was strongly visible on the *l*_o_ domains in the f-PSMs and appeared homogeneous (Fig. 4, *t* = 0 min).

The photo-Gb_3_ is in the *trans-*configuration, and it is expected to behave similarly to a saturated fatty acid-harboring Gb_3_. The observed organization of STxB on the membrane containing the photo-Gb_3_ agrees well with the behavior of membrane-bound STxB reported on phase-separated GUVs (9,54), where STxB is solely bound to the *l*_o_ domains. In rare cases, *l*_o_ domains occurred on s-PSMs to which STxB was also bound (Fig. S7, *white squares*). The *trans-*to-*cis* isomerization of the photo-Gb_3_ upon UV irradiation significantly modified the distribution of STxB on the f-PSMs. Immediately one frame after UV-light irradiation (15 s), a heterogeneous and dynamic protein distribution became discernible, visible as brighter areas surrounded by darker ones where the protein was barely bound (Fig. 4). Simultaneously and similarly to the protein-free samples, *l*_d_ lakes were visible in the f-PSMs. A large portion (∼70%) of the *l*_d_ lakes spontaneously vanished after 10 min, indicating that the phase behavior of the membrane was not significantly altered by the bound protein compared to protein-free f-PSMs (Fig. S6). As the calcein fluorescence remained high while the STxB clusters were formed (Fig. S7), we conclude that the integrity of the f-PSMs is still intact despite the heterogeneity of the bound protein (Video S3).

Video S3. Time-lapse of PSMs with bound STxB after UV and blue light irradiation Frame rate: 20 fps.

Several studies addressed lipid domain remodeling using coexisting *l*_o_/*l*_d_ phase-separated lipid systems combined with light-sensitive probes (23,24,55). However, only a few studies reported on the impact of photo-lipids on protein binding. For example, Carter Ramirez et al. (49) observed cholera toxin B clusters on SLBs doped with the monosialotetrahexosylganglioside G_M1_ after the photo-lysis of caged ceramides. They also observed that the cholera toxin B clusters were still discernible 1 h after UV irradiation. However, these protein clusters were immobile. Since these studies were conducted using SLBs, it is conceivable that the surface properties affected lipid diffusion (17,18), thus preventing the dispersion of protein clusters.

To the best of our knowledge, there is no study on mobile light-induced protein clusters. Here, we observed highly dynamic STxB fluctuations upon isomerization. The *trans*-to-*cis* isomerization of the azobenzene group of Gb_3_ changes the receptor lipid’s geometry. In the *cis*-configuration, the photo-Gb_3_ requires more space, like an unsaturated Gb_3_ molecule, thus altering the organization of STxB on the membrane and leading to the observed compositional fluctuations. As the lateral membrane tension of the PSMs is, with several mN/m, quite large (39,56), the STxB heterogeneity is not a result of membrane undulations (57). The protein density fluctuates on the f-PSMs (Video S4), in contrast to what has been observed for defined STxB clusters on photo-Gb_3_-doped SLBs after UV irradiation (22). In that case, immobile STxB clusters were formed on the *l*_o_ phase. Although the protein density fluctuates over time, protein clusters did not grow, and no stable structures were reached (Video S3). Even blue light irradiation, which should switch the azobenzene moiety from a *cis*- to a *trans*-configuration, did not alter the protein distribution (Fig. 4; Video S4). Glaubitz et al. (13) investigated the influence of the photo-phosphatidylcholine (18-azo-PC) configuration on membrane order and diacylglycerol kinase (Dgka) dynamics. Using lipid vesicles, they noticed a decrease in membrane order and an increase in the mobility of Dgka when 18-azo-PC was in the *cis*-configuration. Consequently, the lack of impact of blue light illumination on protein distribution may be attributed to a higher membrane order (13) and lower lipid diffusion (48) as photo-Gb_3_ goes back to the *trans-*configuration.

Video S4. Zoom in on protein clusters during an illumination cycle Frame rate: 20 fps.

We propose that the dynamic fluctuations in protein density may play a role in the entry process of STxB into host cells. STxB is known to bind up to 15 Gb_3_ molecules, leading to a tight coupling between the protein and the membrane (1). This interaction has been shown to cause lipid compression and reduce the mean molecular area of the lipid bilayer, potentially driving the formation of tubular invaginations that facilitate toxin uptake (7). Recent studies have highlighted the importance of Gb_3_’s acyl chain composition in STxB-induced membrane reorganization. Specifically, Römer et al. (9) found that STxB binding to Gb_3_ with saturated acyl chains (Gb_3_:C_22:0_) did not lead to invaginations, whereas binding to Gb_3_ with unsaturated acyl chains (Gb_3_:C_22:1_) did. Windschiegl et al. (58) further demonstrated that lipid compaction was dependent on the Gb_3_ fatty acid, emphasizing the critical role of Gb_3_ geometry in STxB-induced membrane reorganization.

In this study, we have successfully altered the receptor lipid’s geometry from a *trans*- (geometry of a saturated acyl chain) to a *cis*- (geometry of an unsaturated acyl chain) configuration. This change led to a dynamic, heterogeneous protein distribution on the *l*_o_ phase of the PSMs. The protein arrangements were fully mobile and did not merge into larger clusters over time (Video S4). Although the Gb_3_ concentrations used were unphysiologically high (20 mol %), the observation of mobile, heterogeneous protein distributions in response to Gb_3_ with a larger area demand may help explain why STxB generates invaginations in plasma membranes despite binding to *l*_o_ domains in artificial bilayers, which are energetically more costly to bend than fluid-phase membranes.

## Conclusions

PSMs offer a unique platform for studying the dynamics of lipid domains and protein-induced reorganization processes, which are challenging to investigate with lipid bilayers attached to a solid support. By incorporating the receptor lipid Gb_3_, which contains an azobenzene moiety, into PSMs, we were able to alter the lipid’s geometry in situ using light irradiation. This approach enabled us to examine the dynamic changes in *l*_d_/*l*_o_ phase-separated membranes during the *trans*-to-*cis* isomerization of the photo-receptor, as well as the influence of these geometrical changes on the organization of the membrane-bound protein Shiga toxin. Our results show that light-induced disordered domains quickly disappeared, whereas protein density fluctuations induced by the *trans*-to-*cis* isomerization remained stable, even though they were fully mobile in the freestanding membranes. This suggests that the combination of mobile f-PSMs and azobenzene lipid derivatives can provide a valuable toolkit for investigating the phase behavior of membranes as well as lipid-geometry-sensitive processes.

## Acknowledgments

Shiga toxin B samples were a kind gift from the group of Prof. Dr. Daniel Huster (University of Leipzig). D.B.W. and C.S. thank the 10.13039/501100001659German Research Foundation (SFB 803, Project A05) for financial support. C.S. thanks the Max Planck School Matter to Life (10.13039/501100002347BMBF) for financial support.

## Author contributions

L.S. conceived the study, performed the CLSM experiments, analyzed the CLSM data, and wrote the manuscript. S.A. and D.B.W. designed and performed the synthesis of photo-Gb_3_s and acquired funding. M.S. performed the analysis of domain diffusion. C.S. wrote the manuscript and acquired funding.

## Declaration of interests

The authors declare no competing interests.

## Supporting material

Document S1. Figures S1–S7

Document S2. Article plus supporting material
