# Supplementary material for: Isomerization of photo-Gb3 in phase-separated pore-spanning membranes alters Shiga toxin organization
Source: Biophys J. 2025 Jul 12;124(16):2731–9. doi: 10.1016/j.bpj.2025.07.011 (PMC12414666; doi:10.1016/j.bpj.2025.07.011)
Supplement: Supplementary file 1 [file mmc1.pdf]

Biophysical Journal, Volume *124*

Supplemental information

Isomerization of photo-Gb_3 in phase-separated pore-spanning membranes alters Shiga toxin organization

Larissa Socrier, Somayeh Ahadi, Mark Skamrahl, Daniel B. Werz, and Claudia Steinem

Supplementary information

Isomerization of photoGb$_3$ in phase-separated pore-spanning membranes alters Shiga toxin organization

Larissa Socrier[1,*], Somayeh Ahadi[2], Mark Skamrahl[3], Daniel B. Werz[2,4] and Claudia Steinem[1,3,*]

[1] Max Planck Institute for Dynamics and Self-Organization, Am Faßberg 17, 37077 Göttingen, Germany
[2] Technische Universität Braunschweig, Institute of Organic Chemistry, Hagenring 30, 38106 Braunschweig, Germany
[3] Georg-August-Universität, Institute of Organic and Biomolecular Chemistry, Tammannstraße 2, 37077 Göttingen, Germany
[4] Albert-Ludwigs-Universität Freiburg, Institute of Organic Chemistry, Albertstraße 21, 79104 Freiburg, Germany

Running title: Light-induced STxB clusters in f-PSMs

Correspondance:
Dr. Larissa Socrier
Email: larissa.socrier@ds.mpg.de

Prof. Dr. Claudia Steinem
Email: csteine@gwdg.de

LIST OF SUPPLEMENTARY FIGURES AND MOVIES

FIGURE S1 Selection of PSMs.
FIGURE S2 Irradiation of phase-separated PSMs containing no photo-Gb$_3$.
FIGURE S3 Irradiation of phase-separated PSMs in the presence of photo-Gb$_3$.
FIGURE S4 Influence of the illumination process on the calcein fluorescence in PSMs
FIGURE S5 Selection of PSMs after STxB binding.
FIGURE S6 Quantification of l_d-lakes during the illumination process in presence of STxB.
FIGURE S7 Irradiation of phase-separated PSMs with bound STxB.
MOVIE S1. Time-lapse of control PSMs following UV and blue light irradiation. Frame rate: 20 fps.
MOVIE S2. Time-lapse of photo-Gb$_3$ PSMs following UV and blue light irradiation. Frame rate: 20 fps.
MOVIE S3. Time-lapse of PSMs with bound STxB following UV and blue light irradiation. Frame rate: 20 fps.
MOVIE S4. Zoom in on protein clusters during an illumination cycle. Frame rate: 20 fps.

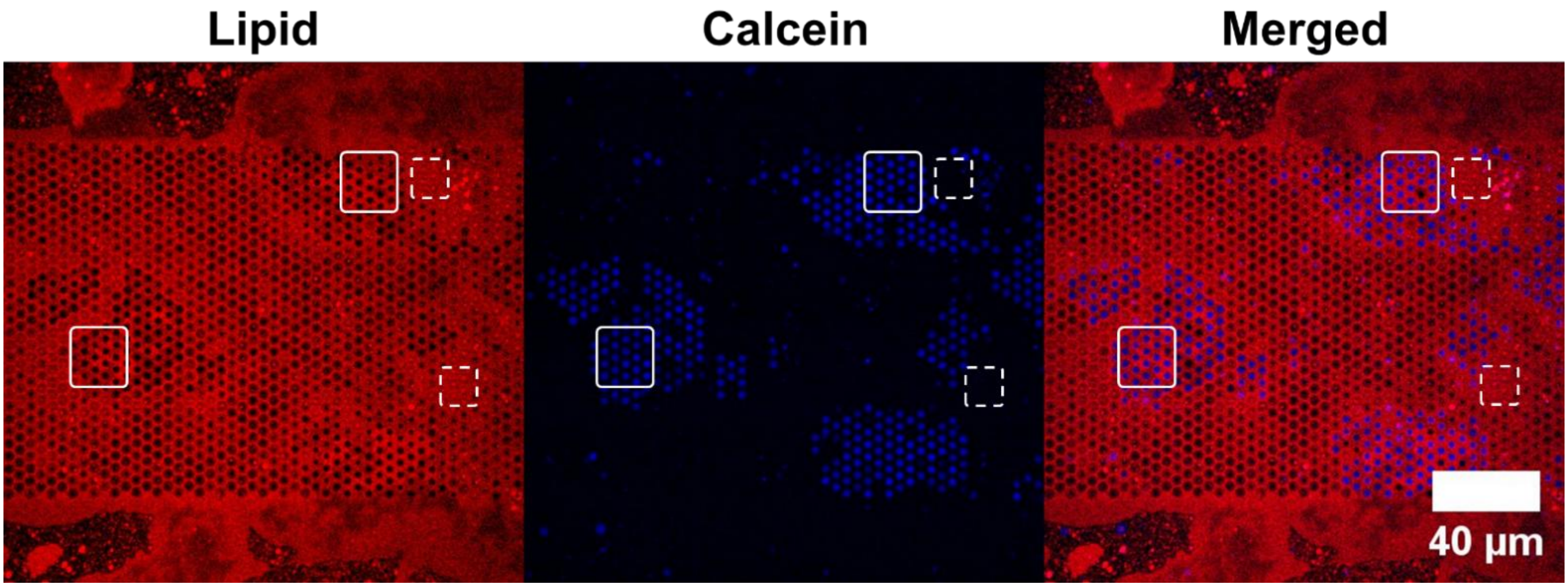

FIGURE S1 Selection of PSMs. Overview of a SiO_x-functionalized porous substrate after GUV sedimentation. Composition: DOPC/SM_{18}/cholesterol/ATTO655-DOPE (39.5:39.5:20:1, *n/n*). The entrapment of the water-soluble dye calcein indicates impermeable f-PSM areas (solid squares) that were chosen for further analysis. If calcein was not entrapped, even if the red fluorescence suggests the formation of membranes (dashed squares), these areas were discarded. A contrast correction factor (γ) of 0.5 was applied for better visualization.

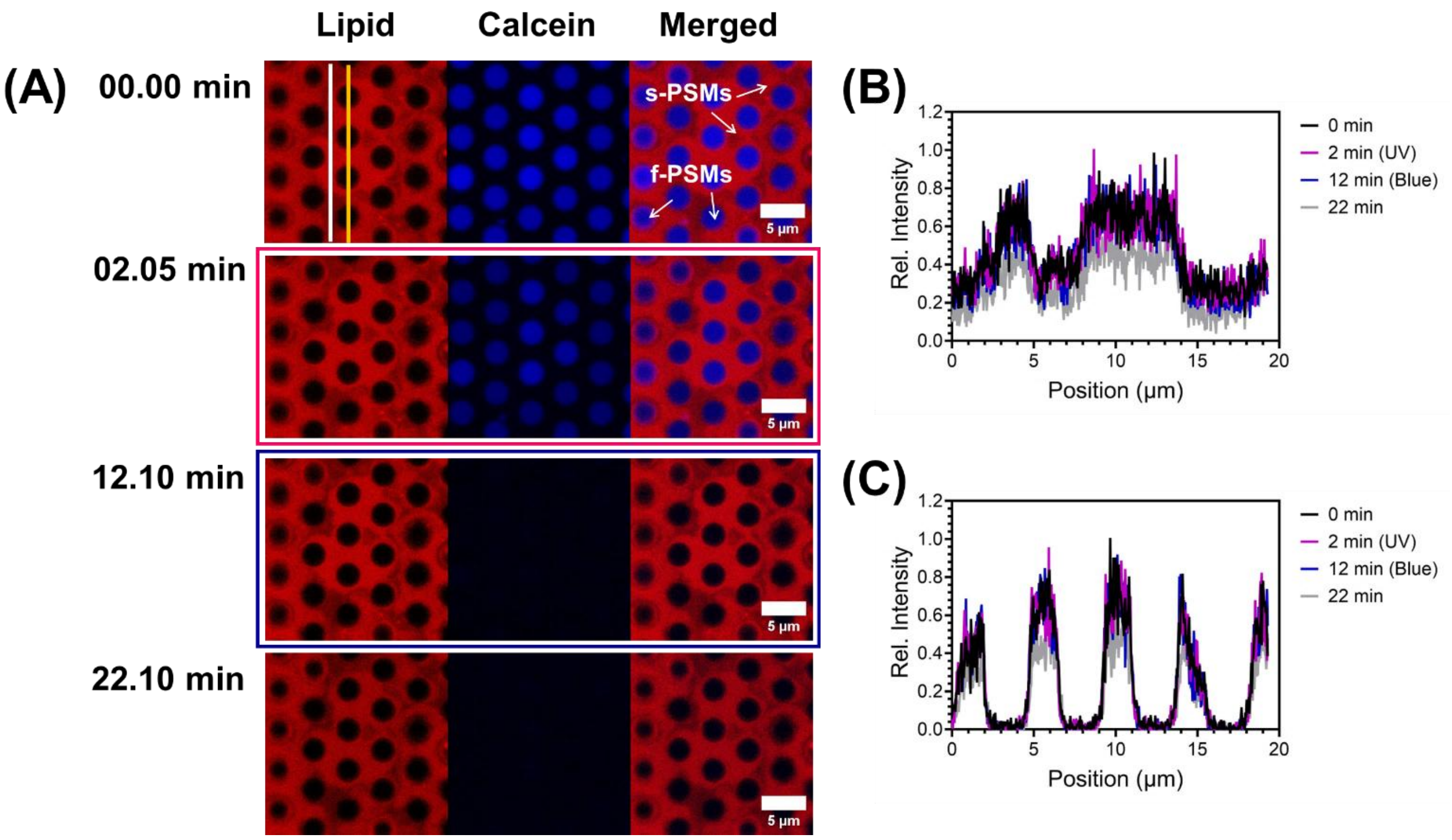

FIGURE S2 Irradiation of phase-separated PSMs containing no photo-Gb_3. Composition: DOPC/SM_{18}/cholesterol/ATTO655-DOPE (39.5:39.5:20:1, *n/n*). (A) A time-lapse was recorded before (black), following UV-irradiation (magenta), blue light irradiation (blue), and after irradiation (grey) (see also Movie S1). A contrast correction factor (γ) of 0.5 was applied for better visualization. (B) Evolution of the fluorescence intensity profile of s-PSMs (white line) and (C) f-PSMs (yellow line) throughout an irradiation cycle.

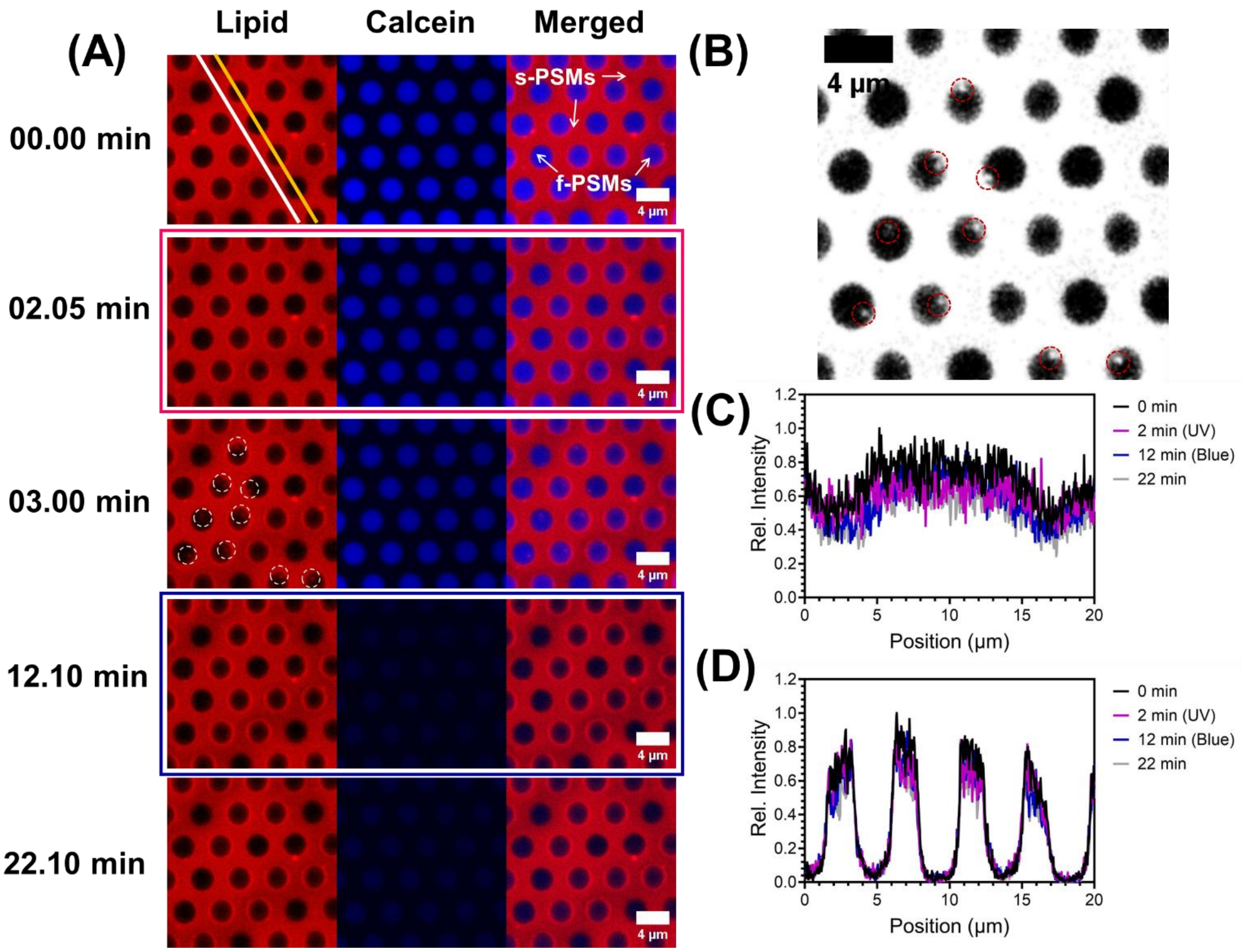

FIGURE S3 Irradiation of phase-separated PSMs in the presence of photo-Gb_3. Lipid composition: DOPC/SM_{18}/cholesterol/photo-Gb_3/ATTO655-DOPE (37:20:22:20:1, *n/n*). (A) Fluorescence images of PSMs (red fluorescence, lipid) and the corresponding calcein fluorescence (blue fluorescence, calcein) and the merged images before irradiation, following exposure to UV light (magenta), blue light (blue) and at the end of the experiment (Movie S2). A contrast correction factor (γ) of 0.5 was applied for better visualization. (B) After Gaussian blurring, a binary image of the lipid channel at 3 min was generated to properly detect l_d-lakes (dashed circles) after UV irradiation. (C) Evolution of the fluorescence intensity profile of s-PSMs (white line) and (D) f-PSMs (yellow line) throughout an irradiation cycle.

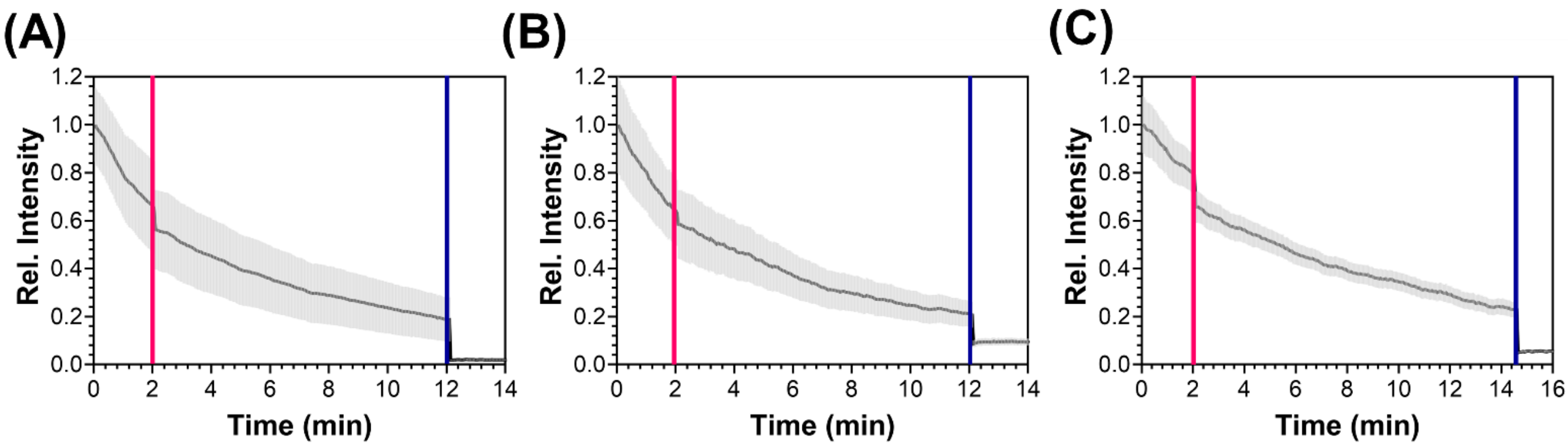

FIGURE S4 Influence of the illumination process on the calcein fluorescence in PSMs. The relative calcein intensity was reported as a function of time during imaging. (A) Control PSMs, *N = 20*. Composition: DOPC/SM_{18}/cholesterol/ATTO655-DOPE (39.5:39.5:20:1, *n/n*). (B) PSMs containing photo-Gb_3, *N = 16*. Composition: DOPC/SM_{18}/cholesterol/photo-Gb_3/ATTO655-DOPE (37:20:22:20:1, *n/n*). (C) Protein-enriched PSMs, *N = 19*. Composition: DOPC/SM_{18}/cholesterol/photo-Gb_3/ATTO655-DOPE (37:20:22:20:1, *n/n*), *c*(STxB) = 300 nM. The vertical lines mark the short irradiation with UV (magenta) and blue light.

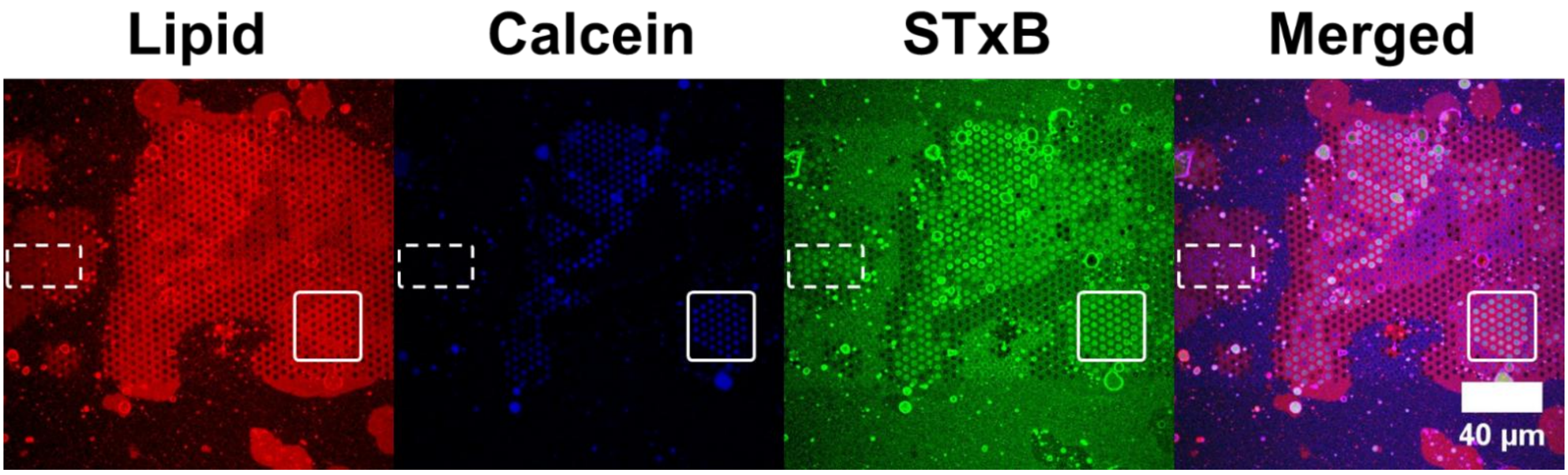

FIGURE S5 Selection of PSMs after STxB binding. Overview of a SiO_x-functionalized porous substrate after GUV sedimentation and incubation with STxB. Composition: DOPC/SM_{18}/cholesterol/photo-Gb_3/ATTO655-DOPE (37:20:22:20:1, *n*/*n*). *c*(STxB) = 300 nM. The entrapment of the water-soluble dye calcein indicates impermeable f-PSM areas (solid squares) that were chosen for further analysis. If calcein was not entrapped, even if the red and green fluorescence suggests the formation of membranes (dashed squares), these areas were discarded. A contrast correction factor (γ) of 0.5 was applied for better visualization.

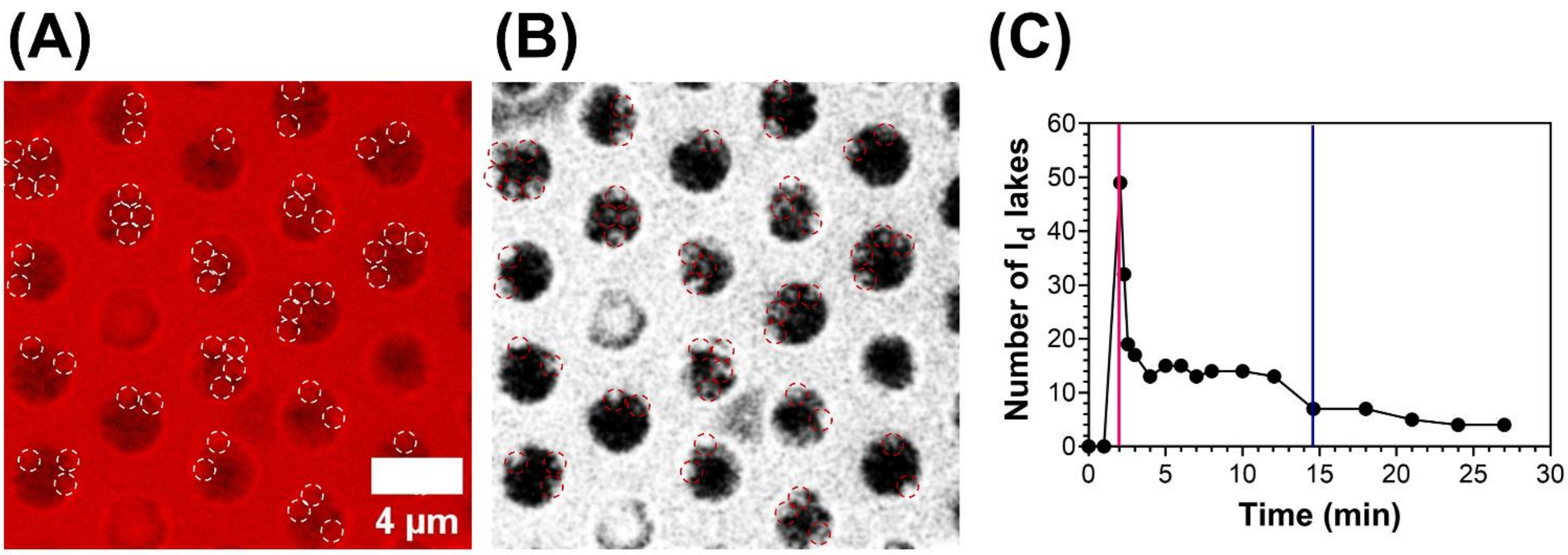

FIGURE S6 Isomerization of photo-Gb_3 in proteo-PSMs. Lipid composition: DOPC/SM_{18}/cholesterol/photo-Gb_3/ATTO655-DOPE (37:20:22:20:1, *n*/*n*). *c*(STxB) = 300 nM. (A) Transient l_d-lakes (white dashed circles) are visible in f-PSMs (dark) 30 s after *trans*-to-*cis* isomerization of photo-Gb_3. (B) For better visualization of the l_d-lakes (red dashed circles) and quantification, a greyscale image was generated with Gaussian blurring. (C) Quantification of l_d-lakes during the illumination process (*N* = 19 f-PSMs). The number of l_d-lakes increased immediately after UV irradiation (magenta line). The l_d-lakes persisted for a few seconds and about 70% of them have vanished after 10 min. A small portion (~10%) of l_d lakes persisted after blue light irradiation (blue line).

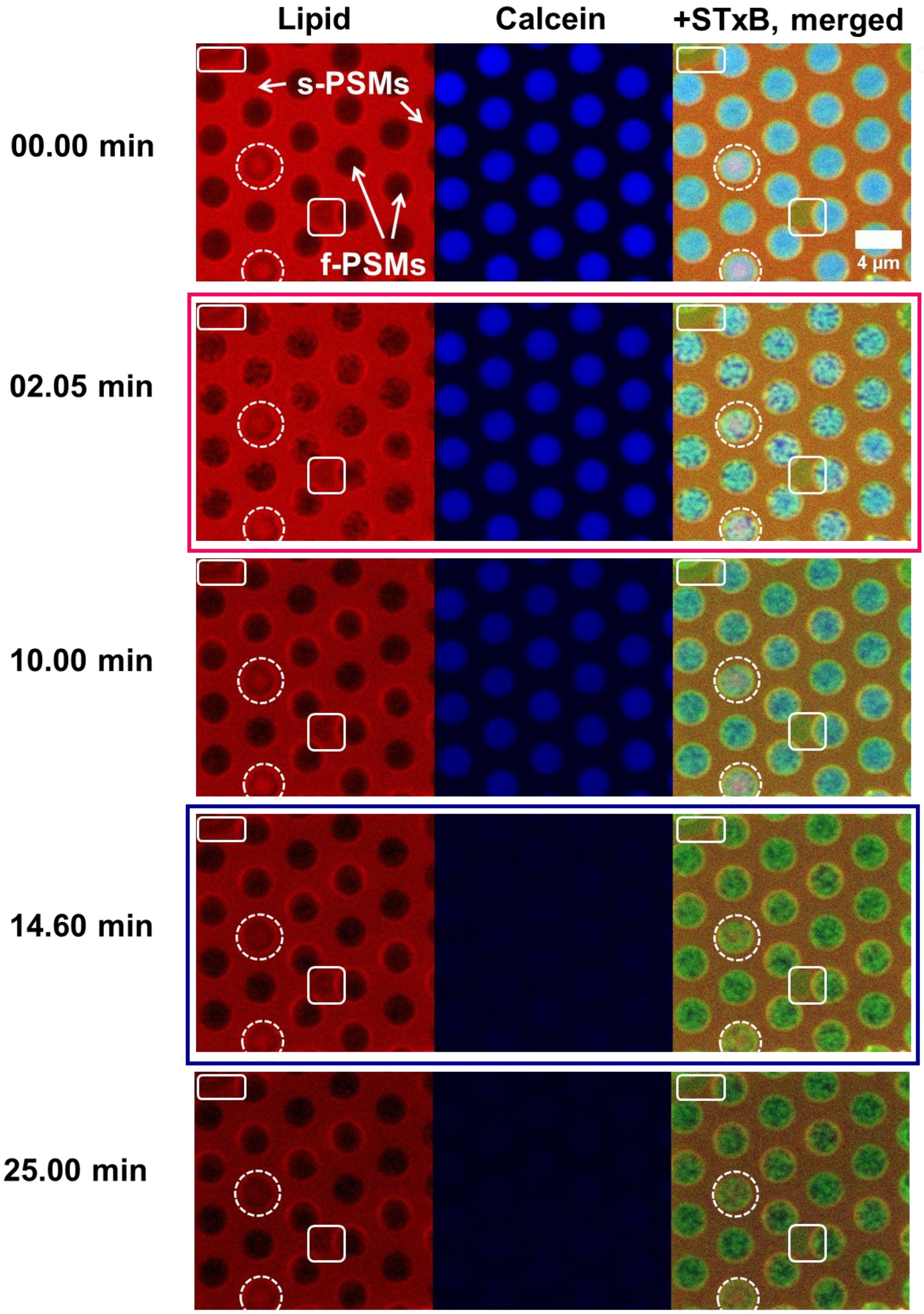

FIGURE S7 Irradiation of phase-separated PSMs with bound STxB. Lipid composition: DOPC/SM_{18}/cholesterol/photo-Gb_3/ATTO655-DOPE, 37:20:22:20:1, *n*/*n*. *c*(STxB) = 300 nM. The l_d-phase, labeled with ATTO655-DOPE (red fluorescence), is located on the s-PSMs, while f-PSMs are composed of the l_o-phase from which the fluorescent dye is excluded. Calcein: The water-soluble dye calcein (blue fluorescence) is entrapped in the micro cups. Merged: All fluorescence intensities are overlaid, including that of STxB (green), which bound to the l_o-phase of the f-PSMs. The white squares highlight a rarely observed ordered domain with bound STxB on an s-PSM. The white circles highlight the rarely observed bend f-PSMs that do not show the characteristic l_o-phase indicated by the exclusion of the lipid dye. A contrast correction factor (γ) of 0.5 was applied for better visualization.
