# Supplementary material for: Isomerization of photo-Gb3 in phase-separated pore-spanning membranes alters Shiga toxin organization
Source: Biophys J. 2025 Jul 12;124(16):2731–9. doi: 10.1016/j.bpj.2025.07.011 (PMC12414666; doi:10.1016/j.bpj.2025.07.011)
Supplement: Supplementary file 2 [file mmc6.pdf]

Biophysical Journal
Article

Biophysical Society

Isomerization of photo-Gb_3 in phase-separated pore-spanning membranes alters Shiga toxin organization

Larissa Socrier,[1,*] Somayeh Ahadi,[2] Mark Skamrahl,[3] Daniel B. Werz,[2,4] and Claudia Steinem[1,3,*]
[1]Max-Planck-Institute for Dynamics and Self-Organization, Göttingen, Germany; [2]Technische Universität Braunschweig, Institute of Organic Chemistry, Braunschweig, Germany; [3]Georg-August-Universität, Institute of Organic and Biomolecular Chemistry, Göttingen, Germany; and [4]Albert-Ludwigs-Universität Freiburg, Institute of Organic Chemistry, Freiburg, Germany

ABSTRACT The lateral organization of Shiga toxin bound to a lipid membrane is significantly influenced by the fatty acid geometry of its receptor glycolipid globotriaosylceramide (Gb_3), which is crucial for the protein's internalization into the host cell. To control the lipid geometry, we used a photoisomerizable azobenzene derivative of Gb_3 (photo-Gb_3) that can be switched between a *trans*- and a *cis*-configuration under light. We reconstituted this photo-Gb_3 into liquid-disordered (l_d)/liquid-ordered (l_o) phase-separated pore-spanning membranes (PSMs), creating freestanding bilayer parts (freestanding PSMs [f-PSMs]) composed of an l_o phase surrounded by an l_d phase on the pore rims. Upon UV irradiation, we observed small, mobile, and transient l_d domains in the f-PSMs, indicating a *trans*-to-*cis* isomerization of the photo-Gb_3. The mean diffusion coefficient of the l_d domains was determined to be 0.014 ± 0.009 $\mu m^2/s$, from which we estimated a membrane surface viscosity of 12–21 $\times 10^{-8}$ Pa s m, indicative of an l_o phase. Before photoisomerization, Shiga toxin B (STxB) bound homogeneously to the l_o phase in the f-PSMs harboring the photo-Gb_3. Upon *trans*-to-*cis* isomerization of the photo-Gb_3, l_d lakes were again formed, and the homogeneous protein distribution turned into dynamic STxB density fluctuations, which are discussed in the context of the entry process of Shiga toxin into the host cell.

SIGNIFICANCE The fatty acid structure of the receptor glycolipid globotriaosylceramide (Gb_3) influences the organization of membrane-bound Shiga toxin, which can impact the entry of the toxin into the host cell. To control the fatty acid geometry of Gb_3 in situ in a time-resolved manner, we employed a Gb_3-azobenzene derivative. Reconstituted into phase-separated pore-spanning membranes, the light-induced *trans*-to-*cis* isomerization of the Gb_3 derivative led to small and mobile liquid-disordered lipid domains and Shiga toxin density fluctuations.

INTRODUCTION

Glycosphingolipids, including Gb_3 (globotriaosylceramide), are a class of lipid molecules known for their significant biological and physiological functions. Found in the outer leaflet of the plasma membrane, they play central roles in various biological processes, such as cell-cell communication, signal transduction, and modulation of membrane biophysical properties. Gb_3, in particular, gains prominence due to its function as a receptor for bacterial Shiga toxins, which are responsible for certain types of food poisoning and other disorders like hemolytic uremic syndrome, colitis, or bloody diarrhea (1,2).

Shiga toxins are AB_5 proteins (3) produced by *Shigella dysenteriae* and enterohemorrhagic *Escherichia coli* and are composed of an enzymatically active A subunit and five B subunits. The B subunits recognize Gb_3 as the first step for the toxin uptake into the host cell. After this uptake, the toxin is translocated to the endoplasmic reticulum via a retrograde transport through the *trans*-Golgi network into the cytosol, where the A_1 domain of the A subunit catalytically removes an adenosine moiety from the 60S ribosomal subunit (4,5).

To investigate the first step of Shiga toxin uptake, model membranes appear well suited (6–8). Using giant unilamellar vesicles (GUVs) and supported lipid bilayers (SLBs) composed of a fluid-phase lipid, sphingomyelin, and cholesterol doped with Gb_3 (9), it was found that Shiga toxin binds solely to the liquid-ordered (l_o) phase in these l_o/liquid-disordered (l_d) phase-separated bilayers. The lateral

Submitted January 20, 2025, and accepted for publication July 10, 2025.
*Correspondence: larissa.socrier@ds.mpg.de or csteine@gwdg.de
Editor: Manuel Prieto.
https://doi.org/10.1016/j.bpj.2025.07.011
© 2025 The Author(s). Published by Elsevier Inc. on behalf of Biophysical Society.
This is an open access article under the CC BY license (http://creativecommons.org/licenses/by/4.0/).

organization of the bound protein is significantly influenced by the Gb_3 geometry. Shiga toxin B (STxB) high-density protein clusters and low-density protein areas were observed on SLBs containing Gb_3 with a saturated $C_{24:0}$ fatty acid (10,11). Such protein clusters, accumulating Gb_3 molecules with a saturated fatty acid, might inhibit membrane bending (9) due to the overall rigid structure of the membrane (12,13). However, a $C_{24:1}$ unsaturated fatty acid leads to protein clusters (14,15) that are separated by protein-free l_o and l_d domains. This protein arrangement is supposed to induce negative curvature, eventually leading to the observed Shiga toxin-induced invaginations (16).

One drawback of SLBs is their direct contact with the support, which reduces the mobility of the lipids and lipid domains (17,18), hampering a rapid reorganization of the membrane and protein arrangement. To overcome this drawback, we developed pore-spanning membranes (PSMs) (19). PSMs are composed of supported membrane regions (supported PSMs [s-PSMs]) and freestanding ones (freestanding PSMs [f-PSMs]), in which lipid domain mobility becomes readily visible on the timescale of milliseconds (20). This membrane system provides the required dynamics (21) to investigate how a change in Gb_3 geometry influences the organization of membrane-bound Shiga toxin in a time-resolved manner.

To switch the Gb_3 geometry, we recently attached an azobenzene group to its fatty acid (22). Photo-switchable lipids, such as azobenzene derivatives, allow the modulation of l_o and l_d domains simply by light (23), which is noninvasive. Upon UV irradiation, the *trans*-to-*cis* isomerization of the azobenzene group leads to a diminution of the area of l_o domains, indicating a reduced membrane order (24). Conversely, after *cis*-to-*trans* isomerization induced by blue light irradiation, the area of l_o domains increases as membrane order is partially restored. Domain rearrangement was found to be dependent on the photo-lipid concentration, the position of the azobenzene group in the hydrophobic core of the membrane (25), and the structure of the polar head of the photo-lipids (24).

Here, we used a synthetic Gb_3 derivative harboring an azobenzene moiety to switch it between the *trans*- and *cis*-configuration, thus altering the geometry of the glycolipid. Employing confocal laser scanning microscopy (CLSM) experiments, we observed changes in membrane organization in the f-PSMs upon light irradiation. The altered Gb_3 lipid geometry also significantly changed the Shiga toxin organization on the l_o phase of the membrane.

MATERIALS AND METHODS

Materials

1,2-dioleoyl-*sn*-glycero-3-phosphocholine (DOPC), *N*-stearoyl-D-erythro-sphingosylphosphoryl-choline ($SM_{18:0}$), cholesterol, and BODIPY-cholesterol were purchased from Avanti Polar Lipids (Alabaster, Alabama). The photo-Gb_3 (Fig. 1 *C*) was synthesized as described previously (22). ATTO655-DOPE was obtained from ATTO Technology (Siegen, Germany) and fluorescein-bis(methyliminodiacetic acid) (calcein) from Merck (Darmstadt, Germany). Silicon monoxide granules (SiO, 4–8 mm) were purchased from Merck. Indium tin oxide coverslips (25×75 mm^2, thickness: 1.1 mm) were obtained from Präzisions Glas & Optik (Iserlohn, Germany). Porous silicon nitride micro-cup substrates with pore diameters of 3 μm were obtained from Aquamarijn Membranes B.V. (Wageningen, the Netherlands). Phosphate-buffered saline (PBS) was prepared using 136 mM NaCl, 2.7 mM KCl, 8.1 mM Na_2HPO_4, and 1.5 mM KH_2PO_4. Calcein-PBS was prepared by dissolving the fluorophore in the buffer to a final concentration of 100 μM. Buffers were adjusted to a pH of 7.4. All buffer reagents were purchased from VWR International (Darmstadt, Germany). Petri dishes (3 cm diameter) were purchased from Mattek (Ashland, Massachusetts).

Preparation of GUVs

GUVs were prepared by electro-formation as described previously (26). Lipids were first dissolved in chloroform and the photo-Gb_3 in chloroform/methanol (2:1, v/v). Mixtures containing DOPC/$SM_{18:0}$/cholesterol/ATTO655-DOPE (39.5:39.5:20:1, *n/n*) or DOPC/$SM_{18:0}$/cholesterol/photo-Gb_3/ATTO655-DOPE (37:20:22:20:1, *n/n*) were prepared in a glass test tube and then spread on indium tin oxide coverslips to form dried lipid films (0.25 mg). The remaining solvent was removed by drying the coverslips under a vacuum at 65°C for 1 h. A homemade chamber was built using a silicon ring and filled with 1.7 mL of sucrose solution (298 mOsm/L). Electro-formation was performed for 3 h by applying an alternating sinusoidal current at a frequency of 12 Hz with an amplitude of 1.6 V (peak to peak). GUVs were collected in black microcentrifuge tubes and conserved for 5 days at room temperature. GUVs containing the chosen ternary lipid mixtures separate into coexisting l_o/l_d phases as the temperature is lowered through the miscibility transition temperature T_{mix}, which is around 40°C.

SiO_x functionalization of porous substrates

The substrates were functionalized as described previously (27). Briefly, porous silicon nitride micro-cup substrates were rinsed with ethanol and ultrapure water and dried with a stream of N_2. The substrates were placed in the center of a convex glass lens beaker, which was subsequently placed in a Bal-TecMed020 high vacuum coating system from Bal-Tec (Balzers, Liechtenstein). SiO was deposited on the substrates at a rate of 0.3 nm/s. Freshly coated substrates were placed in ultrapure water for 1 h at 55°C to form a hydrophilic SiO_x layer (x = 1–2).

Preparation of PSMs

Preparation of the PSMs was carried out in the dark to protect the photo-Gb_3 and at 65°C, well above the miscibility transition temperature T_{mix} of the lipid composition to ensure that the GUVs, spread on the porous support, are homogeneous and in the l_d phase. Freshly coated SiO_x substrates were glued in the center of petri dishes using non-conductive adhesive stickers (Plano, Wetzlar, Germany) and rinsed with ultrapure water. The petri dishes were filled with 3 mL of calcein-containing PBS. 150 μL of the GUV suspension was sedimented on the substrate for 3 min with a 5 mL pipette tip filled with calcein-free PBS. The samples were then slowly cooled to room temperature overnight in the dark. During the cooling process, the PSMs separate into the l_d and l_o phases. Prior to imaging, samples were rinsed abundantly with calcein-free PBS buffer. For protein-binding experiments, STxB was labeled with the fluorophore Cy3 using a fast conjugation kit (ab188287, Abcam, Berlin, Germany). The labeled protein was added to the PSMs at a final concentration of 300 nM. After a gentle agitation for 1 h, the samples were rinsed with calcein-free PBS to remove unbound STxB.

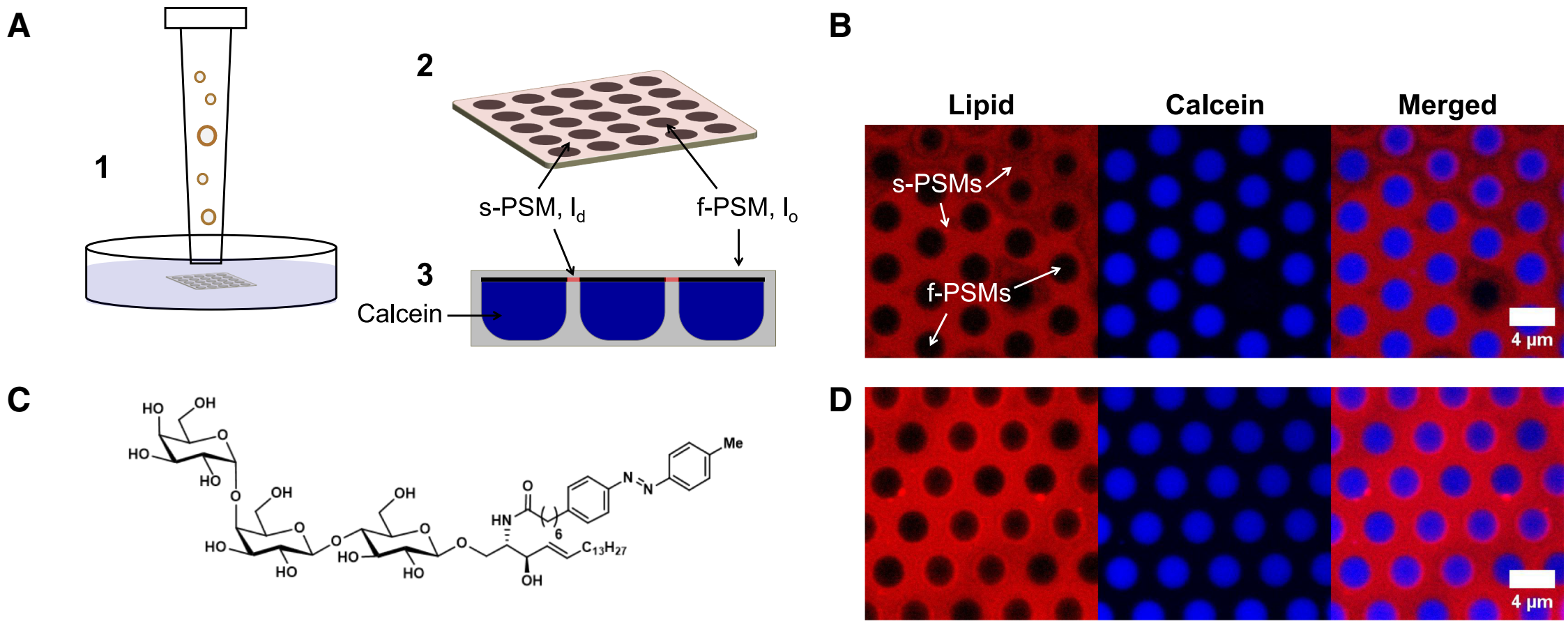

FIGURE 1 (*A*) Preparation of phase-separated pore-spanning membranes. 1) GUVs are sedimented on a SiO_x-functionalized substrate in calcein-containing buffer at 65°C. 2) Samples were cooled down overnight, allowing the formation of l_o domains (*dark*) on the pores and l_d domains (*red*) on the pore rims. 3) Side view of the pores, in which calcein (*blue*) has been entrapped after the spreading of the GUVs, forming PSMs. (*B*) Fluorescence micrographs of phase-separated PSMs on a porous substrate with closed pores at the bottom (micro-cups). Lipid composition: DOPC/SM_{18}/cholesterol/ATTO655-DOPE (39.5:39.5:20:1, *n/n*). Lipid: the l_d phase, labeled with ATTO655-DOPE (*red fluorescence*), is located on the s-PSMs, whereas f-PSMs are composed of the l_o phase from which the fluorescent dye is excluded. Calcein: the water-soluble dye calcein (*blue fluorescence*) is entrapped in the micro-cups. One pore is not covered by a membrane, which appears black. Merged: the lipid dye shows the s-PSMs, whereas the entrapped calcein reports on the pore coverage. In the bottom right corner, the pore is not covered with a membrane, and thus, calcein is not entrapped. (*C*) Chemical structure of photo-Gb_3. (*D*) Fluorescence micrographs of phase-separated PSMs containing photo-Gb_3. Lipid composition: DOPC/SM_{18}/cholesterol/photo-Gb_3/ATTO655-DOPE (37:20:22:20:1, *n/n*). A contrast correction factor (γ) of 0.5 was applied for better visualization.

Light irradiation

A pE-4000 LED from CoolLed (Andover, Hampshire, United Kingdom) was used as a light source to irradiate the samples with UV-A (365 nm) and blue light (435–460 nm). The optical fiber was mounted in the collimator of the microscope so that the light could be directed to the samples via the optical path through the objective. The light power density (in W) applied to the samples was measured using a Field Max II-TO power meter from Coherent (Wilsonville, Oregon). Samples were irradiated for 15 s at a power density of 3 mW/cm^2 with UV light and 150 mW/cm^2 with blue light.

Fluorescence microscopy imaging

CLSM experiments were carried out using an LSM 710 CLSM from Carl Zeiss (Jena, Germany) equipped with a 488 nm Ar laser (1% power), a 633 nm He-Ne laser (3% power), and a 561-10 DPSS laser (3% power) to excite calcein, ATTO655-DOPE, and the Cy3-labeled STxB, respectively. The pinhole was set to 1.5 airy units and the pixel dwell time to 1 μs. Images and time series with 512 $\times$ 512 $pixels^2$ (16-bit resolution) were recorded with a W Plan-Apochromat 63×/1.0 M27 objective. Image analysis was performed with FIJI (http://fiji.sc/Fiji (28)). Graphs were plotted with GraphPad Prism software 10 for Windows (San Diego, California).

Image processing and domain diffusion analysis

To gather information about the diffusion of the observed l_d domains in the f-PSMs, the imaging data were analyzed using self-written Python-based scripts (29–31). Each f-PSM with a visible l_d domain was cropped from the movie with FIJI (28), and the consecutive images in which the l_d domain was visible throughout were subjected to the analysis. At first, a histogram-matching-based adjustment of the image intensities was performed to account for the bleaching of the fluorophore during image acquisition (32). Here, the intensities of all images in each time series were adjusted so that their intensity histogram matched the intensity histogram of the first image as closely as possible. Next, a Gaussian blur was applied to the images to reduce the impact of noise. A two-dimensional Gaussian kernel with a σ value of 70 nm was used for the convolution of all frames. Since the domains only appeared after UV irradiation, the frames before the UV irradiation were used for background correction using the same image-processing routine (cropping out the pore of interest, bleaching correction, and Gaussian blurring). The resulting frames were averaged and then subtracted from each image in which the domain was visible. This increased the contrast between the domains and the background and removed signals from the pore rims. After this preprocessing, a circular mask was applied to the images to exclude image features outside the pore as well as close to the pore rim. A thresholding-based binarization was performed by manually choosing an appropriate intensity threshold to track the domains using the coordinates of the domain centroid. The coordinates of the binarized domain were determined using OpenCV-based contour finding and image moments (33). Plotting was performed using Matplotlib and Trackpy (34,35). The mean-squared displacement (MSD) was calculated from the trajectories, and the diffusion coefficient D was extracted by fitting a linear function to the first four data points of each MSD as $MSD(\tau) = 4D\tau$ with the lag time τ.

RESULTS AND DISCUSSION

Phase behavior of PSMs lacking photo-Gb_3

PSMs were prepared by spreading GUVs composed of DOPC/SM_{18}/cholesterol/ATTO655-DOPE onto SiO_x-coated porous substrates. SiO_x-coated porous substrates are advantageous over gold-covered ones because the intensity of the lipid fluorophores on the supported parts is much less quenched than on gold (20,36). GUV spreading was performed above the miscibility transition temperature of the lipid mixture and in the presence of a calcein-containing buffer (Figs. 1 *A* and S1). After slowly cooling the samples to room temperature overnight,

which resulted in the phase separation of the lipid mixture, fluorescence micrographs were taken. The resulting fluorescence micrographs reveal PSMs comprising freestanding membrane parts (f-PSMs) and supported parts (s-PSMs). The s-PSMs are fluorescent, i.e., they harbor the fluid-phase marker ATTO655-DOPE (Fig. 1 *B*, lipid), indicative of the l_d phase. The fluorescence appears slightly inhomogeneous on the s-PSMs (Figs. 1 *B*, lipid, and S2, *A–C*). The lipid composition, in particular sphingomyelin, can influence the phase behavior of bilayers (37,38); however, the darker regions observed on the s-PSMs might result from an inhomogeneity of the SiO_x functionalization and a varying distance of the s-PSM from the support (39,40). In contrast, the f-PSMs appear dark, as the lipid label is excluded, indicating the l_o phase (39,40).

The observed phase separation of the lipid composition in PSMs agrees with our previous findings (39). In this study, we argued that the re-localization of the fluid-phase lipids onto the pore rims results from the adhesion strength of the lipids to the support (41) and the surface roughness of the SiO_x layer (RMS = 0.49 ± 0.03 nm) (40).

Sumitomo et al. (42) investigated phase-separated PSMs composed of DOPC/DPPC/cholesterol (30:40:30) on SiO_2-coated porous substrates (RMS < 0.3 nm). However, they observed the fluid phase in the f-PSMs, which they partly explained with the overhang at the aperture of the substrate. With a smaller bending rigidity of the l_d phase than the l_o phase, the alignment of the membrane with the substrate topology would energetically be less costly (43). In addition, there is a difference in the overall surface roughness (SiO_2 versus SiO_x) and the lipid composition, with DOPC/DPPC/cholesterol (30:40:30) having an approximately 10°C lower miscibility transition temperature (44).

From the ATTO655-DOPE fluorescence micrographs alone (Fig. 1 *B*, lipid), it is difficult to evaluate whether f-PSMs were formed and whether the PSMs were impermeable. To select the areas on the surface where f-PSMs had been formed, the water-soluble dye calcein was entrapped into the cavities of the micro-cups. From fluorescence overlays of the lipid dye and calcein, the areas with f-PSMs were unambiguously identified (Fig. S1). The observed calcein fluorescence in the cavities clearly shows that f-PSMs with l_o phase, excluding the lipid dye, are present and entrap the water-soluble dye (Fig. 1 *B*, calcein, merged).

Phase behavior and isomerization of Gb_3-containing PSMs

Next, the influence of the photo-Gb_3 on the stability and phase behavior of the PSMs upon isomerization was investigated. PSMs were prepared from GUVs containing 20 mol % of the photo-Gb_3 (DOPC/$SM_{18:0}$/cholesterol/photo-Gb_3/ATTO655-DOPE) using the same protocol. Before irradiation, the fluorescence micrographs of the lipid fluorophore and the calcein were indistinguishable from those without the photo-Gb_3 (Fig. 1 *D*). The PSMs were then exposed to UV-A and blue light irradiation, and the impact of isomerization on the membrane organization was investigated with CLSM. As a control, PSMs lacking the photo-Gb_3 were irradiated (Fig. S2). In the absence of photo-Gb_3, the appearance of the phase-separated PSMs remained unaffected, and no modification of membrane organization became visible (Fig. S2; Video S1). The calcein signal diminished significantly after blue light irradiation, which can be attributed to bleaching (Fig. S4, *A* and *B*), as this fluorophore absorbs in the blue region (45). In the presence of the photo-Gb_3 and before irradiation, the PSMs appear the same as those lacking the azobenzene compound. The PSMs are phase separated, with the l_o phase mainly located on the f-PSMs (Fig. 1 *D*). Upon UV irradiation, small l_d domains became discernible in the f-PSMs (Fig. S3; Video S2). Such domains have been reported previously in SLBs and were termed l_d lakes (24,25). The analysis of the time-dependent calcein fluorescence in the cavities indicates that the f-PSMs did not become leaky upon UV irradiation (Fig. S4 *A* and *B*). Approximately 50% of the f-PSMs displayed at least one l_d lake 60 s after UV irradiation. We assume that the photo-Gb_3 in the *trans*-configuration is preferentially localized in the f-PSMs, which are enriched in l_o-phase lipids. Saturated Gb_3 species were shown to be more miscible with l_o-phase lipids (46), and photo-lipids in the *trans*-configuration behave more like saturated lipids (47). The *trans*-to-*cis* isomerization of the photo-Gb_3 fluidizes small membrane areas leading to l_d lakes. Interestingly, a portion of the l_d lakes disappeared rapidly after UV irradiation. 5 and 10 min after UV illumination, l_d lakes were visible in approximately 30% and 20% of the f-PSMs, respectively (Fig. 2 *C*). The transient nature of the l_d lakes might be a result of the mobility of the lipids in the PSM system. The formed l_d lakes, including the *cis*-configured Gb_3 molecules (48), equilibrate with the l_d phase on the s-PSMs and thus disappear over time (Fig. 2 *C*).

Blue light irradiation led to the complete disappearance of the remaining l_d lakes, suggesting a successful *cis*-to-*trans* isomerization (Fig. 2 *C*; Video S2). On the s-PSMs, the membrane remains in the fluid phase during the entire illumination process (Fig. S3). This observation meets our expectations. s-PSMs are enriched in fluid-phase lipids, and because the *trans*-to-*cis* isomerization leads to fluidization (24,25), the phase does not change. In contrast to the l_d lakes in l_o domains in SLBs after *trans*-to-*cis* isomerization of photo-ceramides (25) and the uncaging of caged ceramides (49), the observed l_d lakes in f-PSMs were fully mobile (Fig. 3). To better visualize these individual l_d domains, we zoomed in on each corresponding f-PSM and applied image processing such as bleaching correction and Gaussian blurring (Fig. 3 *A*). To quantitatively analyze the l_d-lake movement, we used an automated tracking algorithm. An exemplary track of an l_d lake is shown in Fig. 3 *B*. Even though, in this example, an l_d lake was visible for only

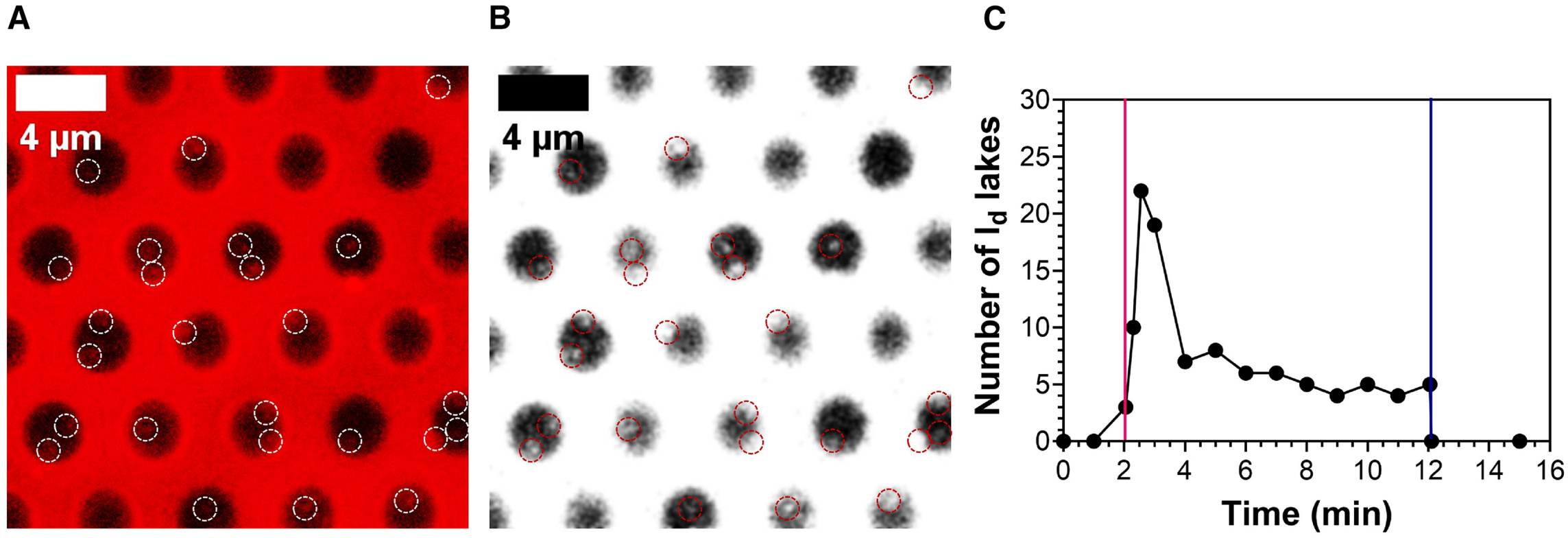

FIGURE 2 Isomerization of photo-Gb_3 in protein-free PSMs. Lipid composition: DOPC/SM_{18}/cholesterol/photo-Gb_3/ATTO655-DOPE, 37:20:22:20:1, *n/n*. (*A*) Transient l_d lakes (*white dashed circles*) are visible in f-PSMs (*dark*) 30 s after *trans*-to-*cis* isomerization of photo-Gb_3. (*B*) For better visualization of the l_d lakes (*red dashed circles*) and quantification, a grayscale image was generated with Gaussian blurring. (*C*) Quantification of l_d lakes during the illumination process ($N = 27$ f-PSMs). The number of l_d lakes increased immediately after UV irradiation (*magenta line*). The lakes persisted for a few seconds and partially vanished over time. Blue light irradiation (*blue line*) led to the complete disappearance of the remaining l_d lakes (Video S2).

87 s, it moved almost across the whole f-PSM. To quantify this diffusion dynamics, we calculated the MSD and used a linear fit to determine the apparent diffusion coefficient of the l_d lake (Fig. 3 *C*).

From the MSD analysis, we calculated a mean diffusion coefficient of $0.014 \pm 0.009\ \mu m^2/s$ (Fig. 3, *C* and *D*). From the obtained diffusion coefficient, one can roughly estimate the membrane surface viscosity using the model of Hughes, Pailthorpe, and White (HPW) (50). Assuming free diffusion of the l_d lakes within the f-PSM, which is a valid assumption given the dimensions of the f-PSM with a radius of $r_{pore} = 1.5\ \mu m$ and a radius of the l_d lakes, which is below the resolution limit of about 400 nm, one can neglect the confinement of the f-PSM as a first approximation (20) and apply the HPW model. They developed a hydrodynamical model applicable under an arbitrary tracer size and for arbitrary membrane viscosities (50). As the HPW model requires complicated numerical computations, Petrov et al. (51,52) proposed a semi-empirical formula describing the HPW model fairly well (Eqs. 1 and 2):

$$D(\lambda) = \frac{k_B T}{4\pi\eta} \frac{\ln(2\lambda) - \gamma + \frac{4}{\pi}\lambda^{-1} - \frac{1}{2}\lambda^{-2}\ln(2\lambda)}{1 - \left(\frac{1}{\pi}\right)\lambda^{-3}\ln(2\lambda) + c_1\lambda^{-b_1} \Big/ \left(1 + c_2\lambda^{-b_2}\right)}, \tag{1}$$

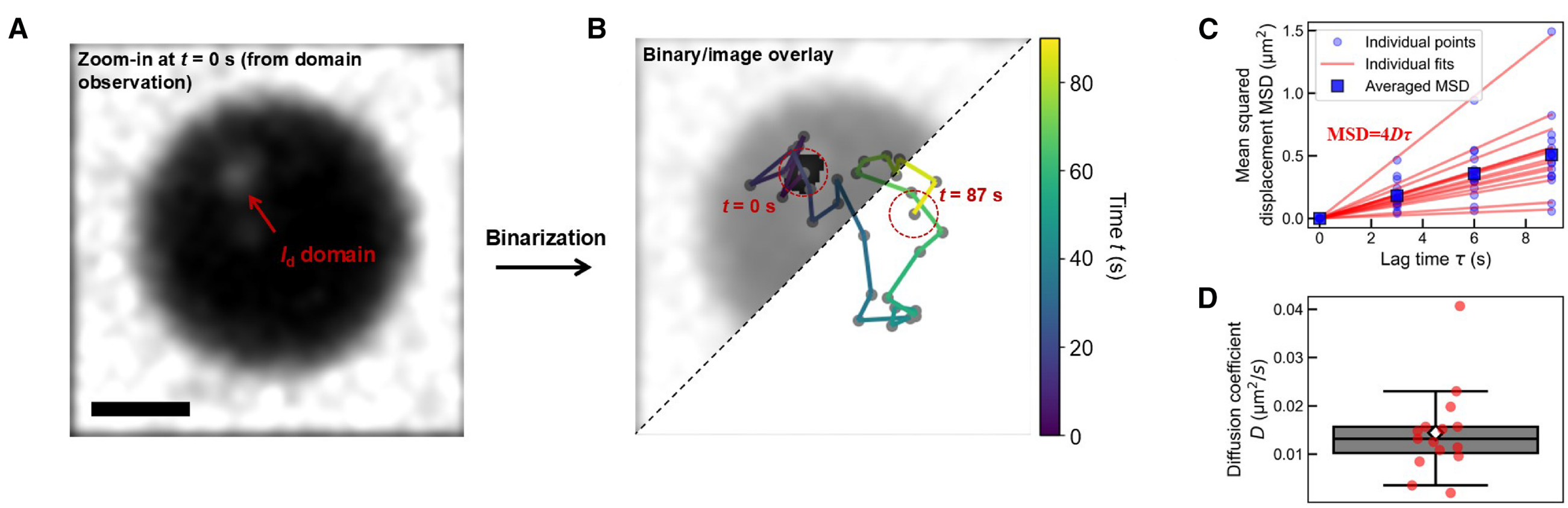

FIGURE 3 Diffusion analysis shows high mobility of the individual domains. (*A*) Greyscale image (after bleaching correction and Gaussian blurring) of an individual f-PSM with a mobile l_d lake (*red arrow*) right after it became visible ($t = 0$ s). Scale bar: 1 μm. (*B*) Overlay of the image from (*A*) (*top left triangle*), the binarized l_d lake (*full image*; *black* corresponds to the recognized domain, whereas *white* was not recognized), and the corresponding trajectory. Red dashed circles indicate the location of the l_d lake at the start point ($t = 0$ s) and the end point ($t = 87$ s) of the trajectory. (*C*) Mean-squared displacement (MSD) curves of all analyzed domains ($n = 15$ single l_d lakes measured across 4 separate experiment days). Individual MSD data points are shown as blue circles. Red lines represent linear fits to the individual MSDs, yielding the diffusion coefficients shown in (*D*) as a boxplot. The box shows the quartiles of the *D* values, and the whiskers correspond to the 5th and 95th percentiles, whereas red circles indicate single measurements. The black line is the median. A mean diffusion coefficient of $0.014 \pm 0.009\ \mu m^2/s$ (*diamond*) was obtained.

with

$$\lambda = \frac{\eta}{2\mu\, r_{\text{lake}}}. \tag{2}$$

η denotes the membrane surface viscosity, μ is the water viscosity, k_B is the Boltzmann constant, T is the temperature, and γ is Euler's constant. The semi-empirically derived constants are as follows: $c_1 = 0.73761$, $c_2 = 0.52119$, $b_1 = 2.74819$, and $b_2 = 0.51465$. Using this model, one can estimate the membrane surface viscosity experienced by the l_d lakes. Assuming radii of the l_d lakes of $r_{\text{lake}} = 10$–400 nm, one obtains membrane surface viscosities of $\eta = 21$–12×10^{-8} Pa s m, which are significantly larger than the values obtained for inclusions in fluid-phase membranes ($\eta = 0.2 \times 10^{-8}$ Pa s m) (51). The large η supports the notion of small l_d lakes diffusing in an l_o phase (53) exhibiting a larger membrane surface viscosity.

Isomerization of protein-enriched PSMs

Based on the mobile f-PSMs with l_o phase, we next addressed how the Gb_3 isomerization influences the lateral organization of membrane-bound STxB. PSMs containing 20 mol % of the photo-Gb_3 (Fig. 1 *C*) were incubated with fluorescently labeled STxB (Fig. S5). The STxB signal was strongly visible on the l_o domains in the f-PSMs and appeared homogeneous (Fig. 4, $t = 0$ min).

The photo-Gb_3 is in the *trans*-configuration, and it is expected to behave similarly to a saturated fatty acid-harboring Gb_3. The observed organization of STxB on the membrane containing the photo-Gb_3 agrees well with the behavior of membrane-bound STxB reported on phase-separated GUVs (9,54), where STxB is solely bound to the l_o domains. In rare cases, l_o domains occurred on s-PSMs to which STxB was also bound (Fig. S7, *white squares*). The *trans*-to-*cis* isomerization of the photo-Gb_3 upon UV irradiation significantly modified the distribution of STxB on the f-PSMs. Immediately one frame after UV-light irradiation (15 s), a heterogeneous and dynamic protein distribution became discernible, visible as brighter areas surrounded by darker ones where the protein was barely bound (Fig. 4). Simultaneously and similarly to the protein-free samples, l_d lakes were visible in the f-PSMs. A large portion (~70%) of the l_d lakes spontaneously vanished after 10 min, indicating that the phase behavior of the membrane was not significantly altered by the bound protein compared to protein-free f-PSMs (Fig. S6). As the calcein fluorescence remained high while the STxB clusters were formed (Fig. S7), we conclude that the integrity of the f-PSMs is still intact despite the heterogeneity of the bound protein (Video S3).

Several studies addressed lipid domain remodeling using coexisting l_o/l_d phase-separated lipid systems combined with light-sensitive probes (23,24,55). However, only a few studies reported on the impact of photo-lipids on protein binding. For example, Carter Ramirez et al. (49) observed cholera toxin B clusters on SLBs doped with the monosialotetrahexosylganglioside G_{M1} after the photo-lysis of caged ceramides. They also observed that the cholera toxin B clusters were still discernible 1 h after UV irradiation. However, these protein clusters were immobile. Since these studies were conducted using SLBs, it is conceivable that the surface properties affected lipid diffusion (17,18), thus preventing the dispersion of protein clusters.

To the best of our knowledge, there is no study on mobile light-induced protein clusters. Here, we observed highly dynamic STxB fluctuations upon isomerization. The *trans*-to-*cis* isomerization of the azobenzene group of Gb_3 changes the receptor lipid's geometry. In the *cis*-configuration, the photo-Gb_3 requires more space, like an unsaturated Gb_3 molecule, thus altering the organization of STxB on the membrane and leading to the observed compositional fluctuations. As the lateral membrane tension of the PSMs is, with several mN/m, quite large (39,56), the STxB heterogeneity is not a result of membrane undulations (57). The protein density fluctuates on the f-PSMs (Video S4), in contrast to what has been observed for defined STxB clusters on photo-Gb_3-doped SLBs after UV irradiation (22). In that case, immobile STxB clusters were formed on the l_o phase. Although the protein density fluctuates over time, protein clusters did not grow, and no stable structures were reached (Video S3). Even blue light irradiation, which should switch the azobenzene moiety from a *cis*- to a *trans*-configuration, did not alter the protein distribution (Fig. 4; Video S4). Glaubitz et al. (13) investigated the influence of the photo-phosphatidylcholine (18-azo-PC) configuration on membrane order and diacylglycerol kinase (Dgka) dynamics. Using lipid vesicles, they noticed a decrease in membrane order and an increase in the mobility of Dgka when 18-azo-PC was in the *cis*-configuration. Consequently, the lack of impact of blue light illumination on protein distribution may be attributed to a higher membrane order (13) and lower lipid diffusion (48) as photo-Gb_3 goes back to the *trans*-configuration.

We propose that the dynamic fluctuations in protein density may play a role in the entry process of STxB into host cells. STxB is known to bind up to 15 Gb_3 molecules, leading to a tight coupling between the protein and the membrane (1). This interaction has been shown to cause lipid compression and reduce the mean molecular area of the lipid bilayer, potentially driving the formation of tubular invaginations that facilitate toxin uptake (7). Recent studies have highlighted the importance of Gb_3's acyl chain composition in STxB-induced membrane reorganization. Specifically, Römer et al. (9) found that STxB binding to Gb_3 with saturated acyl chains (Gb_3:$C_{22:0}$) did not lead to invaginations, whereas binding to Gb_3 with unsaturated acyl chains (Gb_3:$C_{22:1}$) did. Windschiegl et al. (58) further demonstrated that lipid compaction was dependent on the Gb_3 fatty acid, emphasizing the critical role of Gb_3 geometry in STxB-induced membrane reorganization.

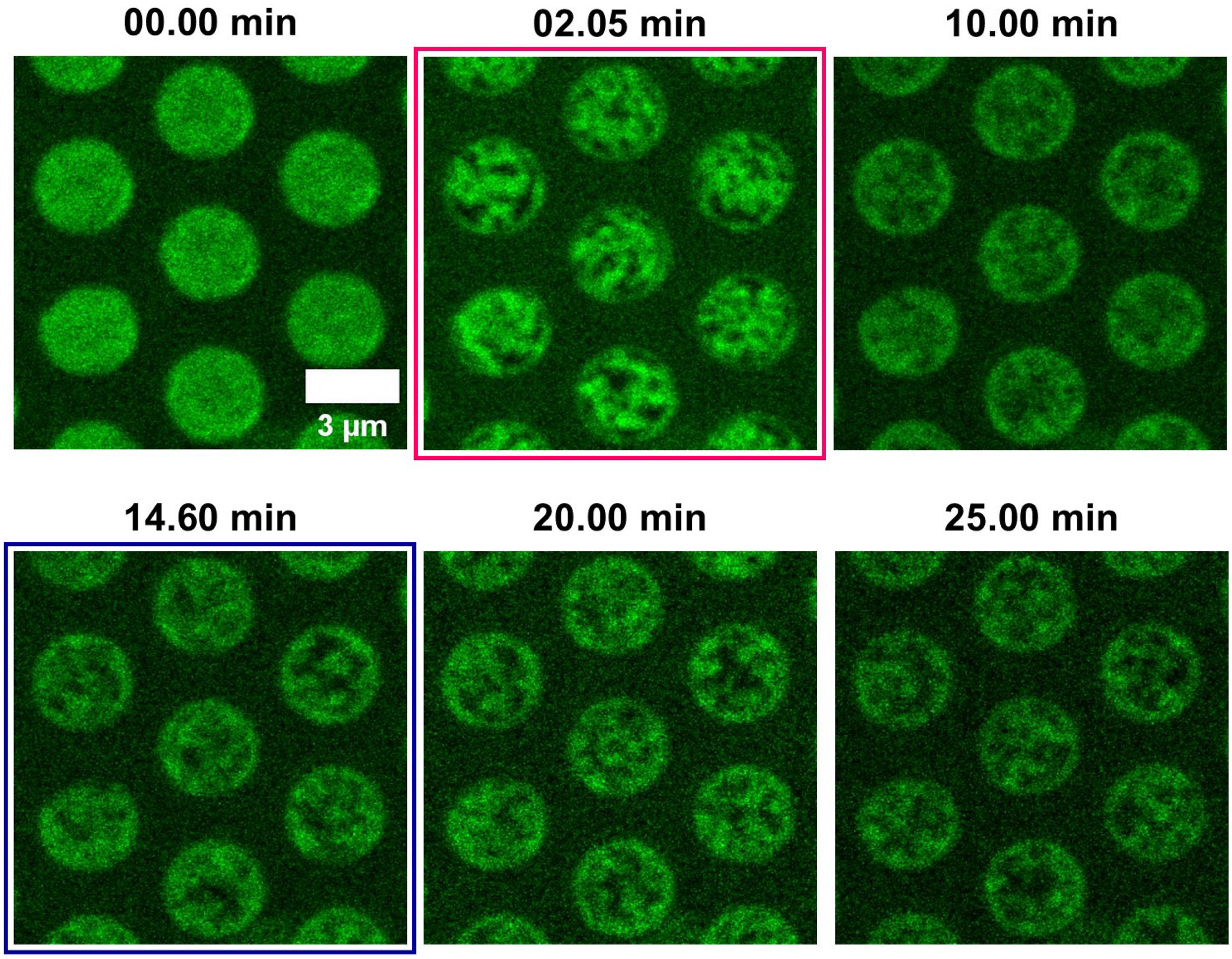

FIGURE 4 Light-induced formation of protein density fluctuations after the isomerization of photo-Gb_3 in f-PSMs. Lipid composition: DOPC/SM_{18}/cholesterol/photo-Gb_3/ATTO655-DOPE, 37:20:22:20:1, *n/n*. *c*(STxB) = 300 nM. A time-lapse was recorded before and after light irradiation (Video S4). Before irradiation, STxB is homogeneously distributed on the surface of the f-PSMs. Protein density fluctuations appeared immediately after UV irradiation (*magenta*) and persisted after blue light irradiation (*blue*).

In this study, we have successfully altered the receptor lipid's geometry from a *trans*- (geometry of a saturated acyl chain) to a *cis*- (geometry of an unsaturated acyl chain) configuration. This change led to a dynamic, heterogeneous protein distribution on the l_o phase of the PSMs. The protein arrangements were fully mobile and did not merge into larger clusters over time (Video S4). Although the Gb_3 concentrations used were unphysiologically high (20 mol %), the observation of mobile, heterogeneous protein distributions in response to Gb_3 with a larger area demand may help explain why STxB generates invaginations in plasma membranes despite binding to l_o domains in artificial bilayers, which are energetically more costly to bend than fluid-phase membranes.

CONCLUSIONS

PSMs offer a unique platform for studying the dynamics of lipid domains and protein-induced reorganization processes, which are challenging to investigate with lipid bilayers attached to a solid support. By incorporating the receptor lipid Gb_3, which contains an azobenzene moiety, into PSMs, we were able to alter the lipid's geometry in situ using light irradiation. This approach enabled us to examine the dynamic changes in l_d/l_o phase-separated membranes during the *trans*-to-*cis* isomerization of the photo-receptor, as well as the influence of these geometrical changes on the organization of the membrane-bound protein Shiga toxin. Our results show that light-induced disordered domains quickly disappeared, whereas protein density fluctuations induced by the *trans*-to-*cis* isomerization remained stable, even though they were fully mobile in the freestanding membranes. This suggests that the combination of mobile f-PSMs and azobenzene lipid derivatives can provide a valuable toolkit for investigating the phase behavior of membranes as well as lipid-geometry-sensitive processes.

ACKNOWLEDGMENTS

Shiga toxin B samples were a kind gift from the group of Prof. Dr. Daniel Huster (University of Leipzig). D.B.W. and C.S. thank the German Research Foundation (SFB 803, Project A05) for financial support. C.S. thanks the Max Planck School Matter to Life (BMBF) for financial support.

AUTHOR CONTRIBUTIONS

L.S. conceived the study, performed the CLSM experiments, analyzed the CLSM data, and wrote the manuscript. S.A. and D.B.W. designed and performed the synthesis of photo-Gb_3s and acquired funding. M.S. performed the analysis of domain diffusion. C.S. wrote the manuscript and acquired funding.

DECLARATION OF INTERESTS

The authors declare no competing interests.

SUPPORTING MATERIAL

Supporting material can be found online at https://doi.org/10.1016/j.bpj.2025.07.011.

REFERENCES

1. Bergan, J., A. B. Dyve Lingelem, K. Sandvig…, 2012. Shiga toxins. *Toxicon.* 60:1085–1107.
2. Melton-Celsa, A. R. 2014. Shiga Toxin (Stx) Classification, Structure, and Function. *Microbiol. Spectr.* 2:EHEC-0024-2013.
3. Brown, P. I., A. Ojiakor, C. C. Fowler…, 2023. The diverse landscape of AB_5-type toxins. *Eng. Microbiol.* 3:100104.
4. Beddoe, T., A. W. Paton, …, J. C. Paton. 2010. Structure, biological functions and applications of the AB5 toxins. *Trends Biochem. Sci.* 35:411–418.
5. Sandvig, K., J. Bergan, …, T. Skotland. 2014. Lipid requirements for entry of protein toxins into cells. *Prog. Lipid Res.* 54:1–13.
6. Wang, R., J. Shi, B. I. Swanson…, 2004. Evidence for cholera aggregation on G_{M1}-decorated lipid bilayers. *Colloids Surf. B Biointerfaces.* 33:45–51.
7. Watkins, E. B., J. Majewski, …, L. Johannes. 2019. Shiga Toxin Induces Lipid Compression: A Mechanism for Generating Membrane Curvature. *Nano Lett.* 19:7365–7369.
8. Schubert, T., T. Sych, …, W. Römer. 2020. Differential recognition of lipid domains by two Gb3-binding lectins. *Sci. Rep.* 10:9752.
9. Römer, W., L. Berland, …, L. Johannes. 2007. Shiga toxin induces tubular membrane invaginations for its uptake into cells. *Nature.* 450:670–675.
10. Schütte, O. M., A. Ries, D. B. Werz…, 2014. Influence of Gb_3 glycosphingolipids differing in their fatty acid chain on the phase behaviour of solid supported membranes: chemical syntheses and impact of Shiga toxin binding. *Chem. Sci.* 5:3104–3114.
11. Sibold, J., S. Ahadi, C. Steinem…, 2021. Chemically synthesized Gb_3 glycosphingolipids: tools to access their function in lipid membranes. *Eur. Biophys. J.* 50:109–126.
12. Watkins, E. B., H. Gao, L. Johannes…, 2014. Carbohydrate conformation and lipid condensation in monolayers containing glycosphingolipid Gb_3: influence of acyl chain structure. *Biophys. J.* 107:1146–1155.
13. Doroudgar, M., J. Morstein, …, C. Glaubitz. 2021. How Photoswitchable Lipids Affect the Order and Dynamics of Lipid Bilayers and Embedded Proteins. *J. Am. Chem. Soc.* 143:9515–9528.
14. Gracià, R. S., N. Bezlyepkina, …, R. Dimova. 2010. Effect of cholesterol on the rigidity of saturated and unsaturated membranes: Fluctuation and electrodeformation analysis of giant vesicles. *Soft Matter.* 6:1472–1482.
15. Kollmitzer, B., P. Heftberger, …, G. Pabst. 2013. Monolayer spontaneous curvature of raft-forming membrane lipids. *Soft Matter.* 9:10877–10884.
16. Pezeshkian, W., A. G. Hansen, …, J. H. Ipsen. 2016. Membrane invagination induced by Shiga toxin B-subunit: From molecular structure to tube formation. *Soft Matter.* 12:5164–5171.
17. Przybylo, M., J. Sýkora, …, M. Hof. 2006. Lipid Diffusion in Giant Unilamellar Vesicles Is More than 2 Times Faster than in Supported Phospholipid Bilayers under Identical Conditions. *Langmuir.* 22:9096–9099.
18. Seu, K. J., A. P. Pandey, …, J. S. Hovis. 2007. Effect of surface treatment on diffusion and domain formation in supported lipid bilayers. *Biophys. J.* 92:2445–2450.
19. Kocun, M., T. D. Lazzara, …, A. Janshoff. 2011. Preparation of solvent-free, pore-spanning lipid bilayers: Modeling the low tension of plasma membranes. *Langmuir.* 27:7672–7680.
20. Schütte, O. M., I. Mey, …, C. Steinem. 2017. Size and mobility of lipid domains tuned by geometrical constraints. *Proc. Natl. Acad. Sci. USA.* 114:E6064–E6071.
21. Spindler, S., J. Sibold, …, V. Sandoghdar. 2018. High-Speed Microscopy of Diffusion in Pore-Spanning Lipid Membranes. *Nano Lett.* 18:5262–5271.
22. Socrier, L., S. Ahadi, C. Steinem…, 2023. Optical manipulation of Gb_3 enriched lipid domains: impact of isomerization on Gb_3 -Shiga toxin B interaction. *Chem. Eur. J.* 29:e202202766.
23. Socrier, L., and C. Steinem. 2023. Photo-Lipids: Light-Sensitive Nano-Switches to Control Membrane Properties. *ChemPlusChem.* 88:e202300203.
24. Hartrampf, N., S. M. Leitao, …, H. G. Franquelim. 2023. Structural diversity of photoswitchable sphingolipids for optodynamic control of lipid microdomains. *Biophys. J.* 122:2325–2341.
25. Frank, J. A., H. G. Franquelim, …, D. Trauner. 2016. Optical Control of Lipid Rafts with Photoswitchable Ceramides. *J. Am. Chem. Soc.* 138:12981–12986.
26. Socrier, L., A. Sharma, C. Steinem…, 2023. Fluorophore position of headgroup-labeled Gb_3 glycosphingolipids in lipid bilayers. *Biophys. J.* 122:4104–4112.
27. Socrier, L., and C. Steinem. 2024. Pore-spanning membranes as a tool to investigate lateral lipid membrane heterogeneity. *In* Methods in Enzymology. T. Baumgart and M. Deserno, eds Elsevier, pp. 455–483.
28. Schindelin, J., I. Arganda-Carreras, …, A. Cardona. 2012. Fiji: An open-source platform for biological-image analysis. *Nat. Methods.* 9:676–682.
29. Harris, C. R., K. J. Millman, …, T. E. Oliphant. 2020. Array programming with NumPy. *Nature.* 585:357–362.
30. Virtanen, P., R. Gommers, …, Y. Vázquez-Baeza. 2020. SciPy 1.0: fundamental algorithms for scientific computing in Python. *Nat. Methods.* 17:261–272.
31. McKinney, W. 2010. Data Structures for Statistical Computing in Python. *In* Proceedings of the 9th Python in Science Conference, pp. 56–61.
32. van der Walt, S., J. L. Schönberger; …, scikit-image contributors. 2014. scikit-image: image processing in Python. *PeerJ.* 2:e453.
33. Bradski, G. 2000. The OpenCV Library. *Dr. Dobb's J. Softw. Tools Prof. Program.* 25:120–125.
34. Hunter, J. D. 2007. Matplotlib: A 2D Graphics Environment. *Comput. Sci. Eng.* 9:90–95.
35. Crocker, J. C., and D. G. Grier. 1996. Methods of Digital Video Microscopy for Colloidal Studies. *J. Colloid Interface Sci.* 179:298–310.
36. Kramer, K., M. Sari, …, C. Steinem. 2022. From LUVs to GUVs—How to Cover Micrometer-Sized Pores with Membranes. *J. Phys. Chem. B.* 126:8233–8244.
37. Jiménez-Rojo, N., A. B. García-Arribas, …, F. M. Goñi. 2014. Lipid bilayers containing sphingomyelins and ceramides of varying N-acyl lengths: A glimpse into sphingolipid complexity. *Biochim. Biophys. Acta Biomembr.* 1838:456–464.

38. Goñi, F. M., and A. Alonso. 2006. Biophysics of sphingolipids I. Membrane properties of sphingosine, ceramides and other simple sphingolipids. *Biochim. Biophys. Acta Biomembr.* 1758:1902–1921.

39. Sibold, J., V. E. Tewaag, …, C. Steinem. 2020. Phase separation in pore-spanning membranes induced by differences in surface adhesion. *Phys. Chem. Chem. Phys.* 22:9308–9315.

40. Teske, N., J. Sibold, …, C. Steinem. 2017. Continuous Pore-Spanning Lipid Bilayers on Silicon Oxide-Coated Porous Substrates. *Langmuir.* 33:14175–14183.

41. Kuhlmann, J. W., I. P. Mey, and C. Steinem. 2014. Modulating the lateral tension of solvent-free pore-spanning membranes. *Langmuir.* 30:8186–8192.

42. Sumitomo, K., and A. Oshima. 2017. Liquid-Ordered/Liquid-Crystalline Phase Separation at a Lipid Bilayer Suspended over Microwells. *Langmuir.* 33:13277–13283.

43. Böcker, M., S. Muschter, …, T. E. Schäffer. 2009. Imaging and patterning of pore-suspending membranes with scanning ion conductance microscopy. *Langmuir.* 25:3022–3028.

44. Veatch, S. L., and S. L. Keller. 2005. Miscibility phase diagrams of giant vesicles containing sphingomyelin. *Phys. Rev. Lett.* 94:148101.

45. Kitchen, P., M. M. Salman, …, R. M. Bill. 2020. Calcein Fluorescence Quenching to Measure Plasma Membrane Water Flux in Live Mammalian Cells. *STAR Protoc.* 1:100157.

46. Socrier, L., C. Bail, C. Steinem…, 2022. The interaction of Gb_3 glycosphingolipids with l_d and l_o phase lipids in lipid monolayers is a function of their fatty acids. *Langmuir.* 38:5874–5882.

47. Urban, P., S. D. Pritzl, …, T. Lohmüller. 2018. Light-Controlled Lipid Interaction and Membrane Organization in Photolipid Bilayer Vesicles. *Langmuir.* 34:13368–13374.

48. Urban, P., S. D. Pritzl, T. Lohmüller…, 2020. A Lipid Photoswitch Controls Fluidity in Supported Bilayer Membranes. *Langmuir.* 36:2629–2634.

49. Carter Ramirez, D. M., Y. A. Kim, …, L. J. Johnston. 2013. Lipid phase separation and protein-ganglioside clustering in supported bilayers are induced by photorelease of ceramide. *Soft Matter.* 9:4890–4899.

50. Hughes, B. D., B. A. Pailthorpe, and L. R. White. 1981. The translational and rotational drag on a cylinder moving in a membrane. *J. Fluid Mech.* 110:349–372.

51. Petrov, E. P., R. Petrosyan, and P. Schwille. 2012. Translational and rotational diffusion of micrometer-sized solid domains in lipid membranes. *Soft Matter.* 8:7552.

52. Petrov, E. P., and P. Schwille. 2008. Translational Diffusion in Lipid Membranes beyond the Saffman-Delbrück Approximation. *Biophys. J.* 94:L41–L43.

53. Sakuma, Y., T. Kawakatsu, …, M. Imai. 2020. Viscosity Landscape of Phase-Separated Lipid Membrane Estimated from Fluid Velocity Field. *Biophys. J.* 118:1576–1587.

54. Safouane, M., L. Berland, …, P. Bassereau. 2010. Lipid Cosorting Mediated by Shiga Toxin Induced Tubulation. *Traffic.* 11:1519–1529.

55. Saitov, A., S. A. Akimov, …, P. Pohl. 2020. Ordered Lipid Domains Assemble via Concerted Recruitment of Constituents from Both Membrane Leaflets. *Phys. Rev. Lett.* 124:108102.

56. Mey, I., M. Stephan, …, A. Janshoff. 2009. Local membrane mechanics of pore-spanning bilayers. *J. Am. Chem. Soc.* 131:7031–7039.

57. Pezeshkian, W., H. Gao, …, J. C. Shillcock. 2017. Mechanism of Shiga Toxin Clustering on Membranes. *ACS Nano.* 11:314–324.

58. Windschiegl, B., A. Orth, C. Steinem…, 2009. Lipid reorganization induced by Shiga toxin clustering on planar membranes. *PLoS One.* 4:1–11.

Biophysical Journal, Volume *124*

Supplemental information

Isomerization of photo-Gb_3 in phase-separated pore-spanning membranes alters Shiga toxin organization

Larissa Socrier, Somayeh Ahadi, Mark Skamrahl, Daniel B. Werz, and Claudia Steinem

Supplementary information

Isomerization of photoGb$_3$ in phase-separated pore-spanning membranes alters Shiga toxin organization

Larissa Socrier[1,*], Somayeh Ahadi[2], Mark Skamrahl[3], Daniel B. Werz[2,4] and Claudia Steinem[1,3,*]

[1] Max Planck Institute for Dynamics and Self-Organization, Am Faßberg 17, 37077 Göttingen, Germany

[2] Technische Universität Braunschweig, Institute of Organic Chemistry, Hagenring 30, 38106 Braunschweig, Germany

[3] Georg-August-Universität, Institute of Organic and Biomolecular Chemistry, Tammannstraße 2, 37077 Göttingen, Germany

[4] Albert-Ludwigs-Universität Freiburg, Institute of Organic Chemistry, Albertstraße 21, 79104 Freiburg, Germany

Running title: Light-induced STxB clusters in f-PSMs

Correspondance:
Dr. Larissa Socrier
Email: larissa.socrier@ds.mpg.de

Prof. Dr. Claudia Steinem
Email: csteine@gwdg.de

LIST OF SUPPLEMENTARY FIGURES AND MOVIES

FIGURE S1 Selection of PSMs.
FIGURE S2 Irradiation of phase-separated PSMs containing no photo-Gb$_3$.
FIGURE S3 Irradiation of phase-separated PSMs in the presence of photo-Gb$_3$.
FIGURE S4 Influence of the illumination process on the calcein fluorescence in PSMs
FIGURE S5 Selection of PSMs after STxB binding.
FIGURE S6 Quantification of l_d-lakes during the illumination process in presence of STxB.
FIGURE S7 Irradiation of phase-separated PSMs with bound STxB.
MOVIE S1. Time-lapse of control PSMs following UV and blue light irradiation. Frame rate: 20 fps.
MOVIE S2. Time-lapse of photo-Gb$_3$ PSMs following UV and blue light irradiation. Frame rate: 20 fps.
MOVIE S3. Time-lapse of PSMs with bound STxB following UV and blue light irradiation. Frame rate: 20 fps.
MOVIE S4. Zoom in on protein clusters during an illumination cycle. Frame rate: 20 fps.

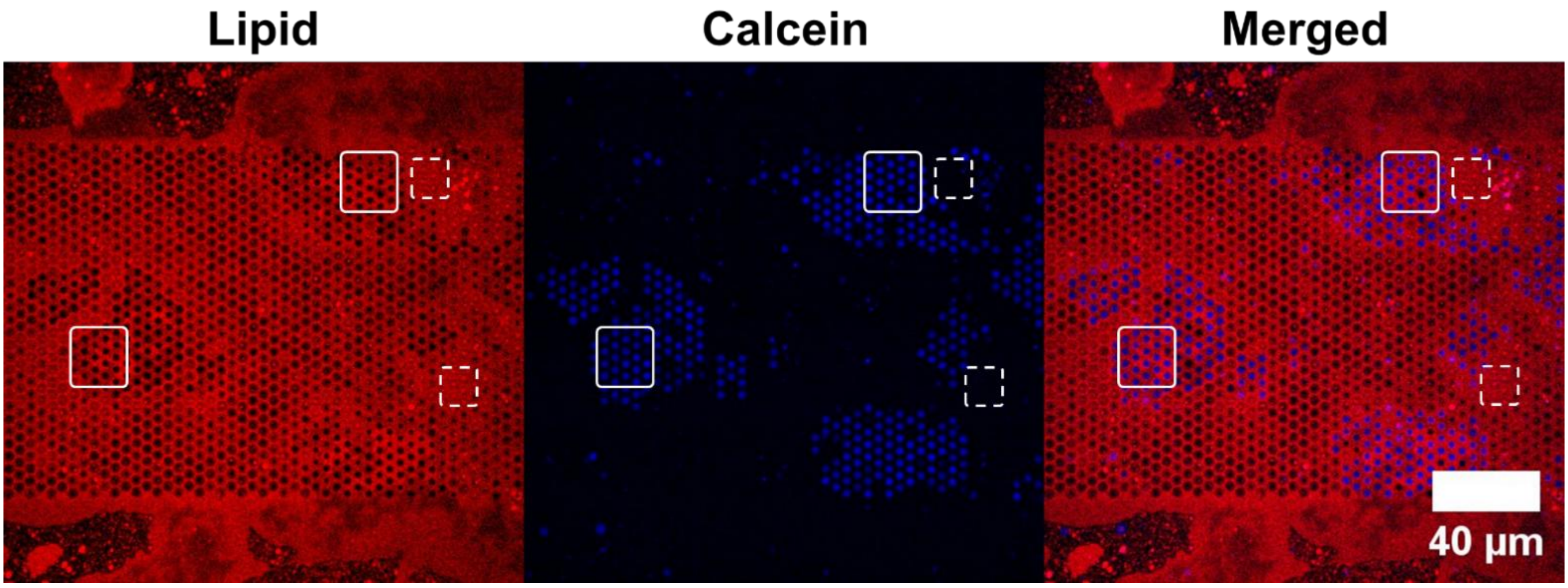

FIGURE S1 Selection of PSMs. Overview of a SiO_x-functionalized porous substrate after GUV sedimentation. Composition: DOPC/SM_{18}/cholesterol/ATTO655-DOPE (39.5:39.5:20:1, *n/n*). The entrapment of the water-soluble dye calcein indicates impermeable f-PSM areas (solid squares) that were chosen for further analysis. If calcein was not entrapped, even if the red fluorescence suggests the formation of membranes (dashed squares), these areas were discarded. A contrast correction factor (γ) of 0.5 was applied for better visualization.

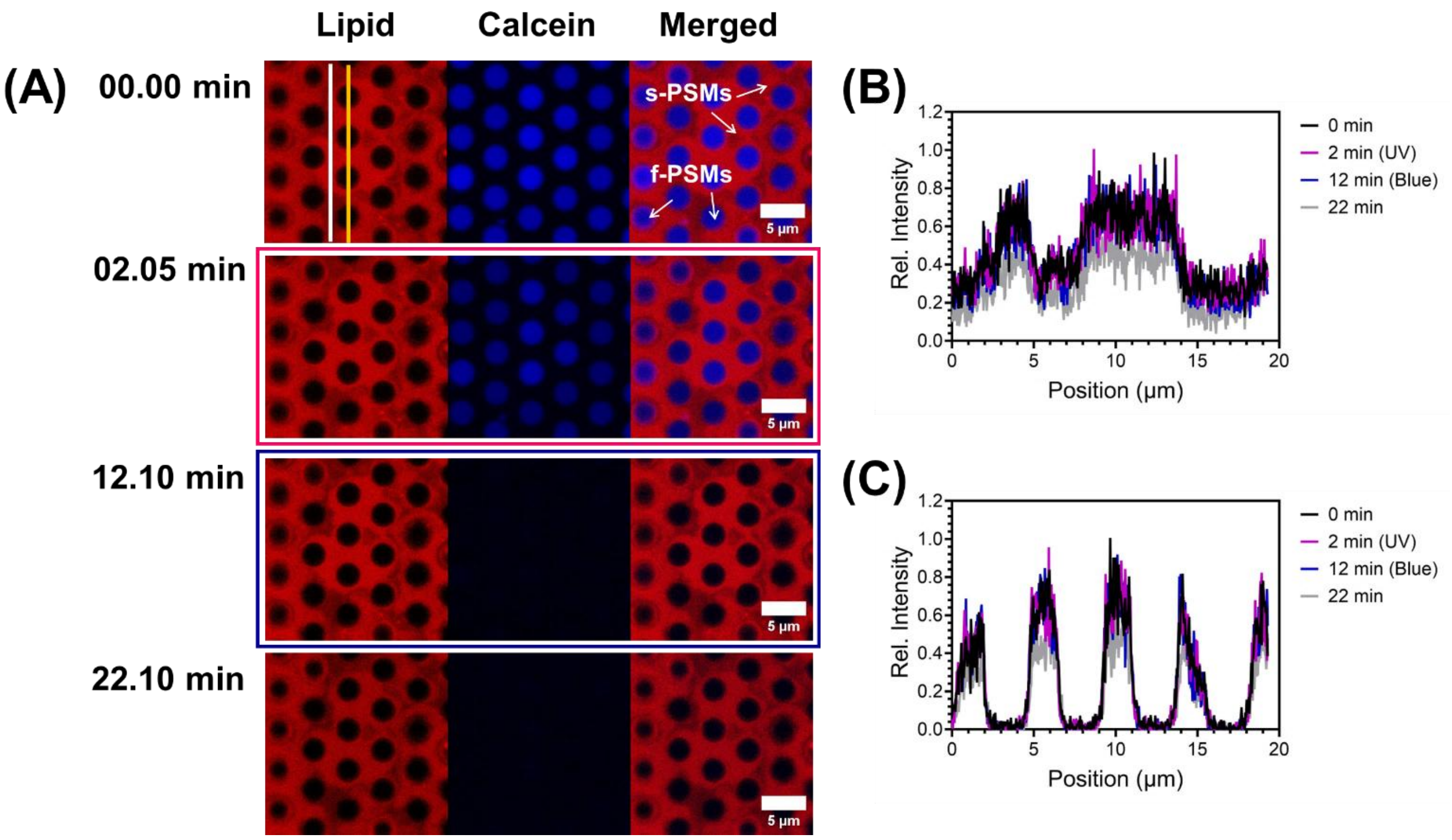

FIGURE S2 Irradiation of phase-separated PSMs containing no photo-Gb_3. Composition: DOPC/SM_{18}/cholesterol/ATTO655-DOPE (39.5:39.5:20:1, *n/n*). (A) A time-lapse was recorded before (black), following UV-irradiation (magenta), blue light irradiation (blue), and after irradiation (grey) (see also Movie S1). A contrast correction factor (γ) of 0.5 was applied for better visualization. (B) Evolution of the fluorescence intensity profile of s-PSMs (white line) and (C) f-PSMs (yellow line) throughout an irradiation cycle.

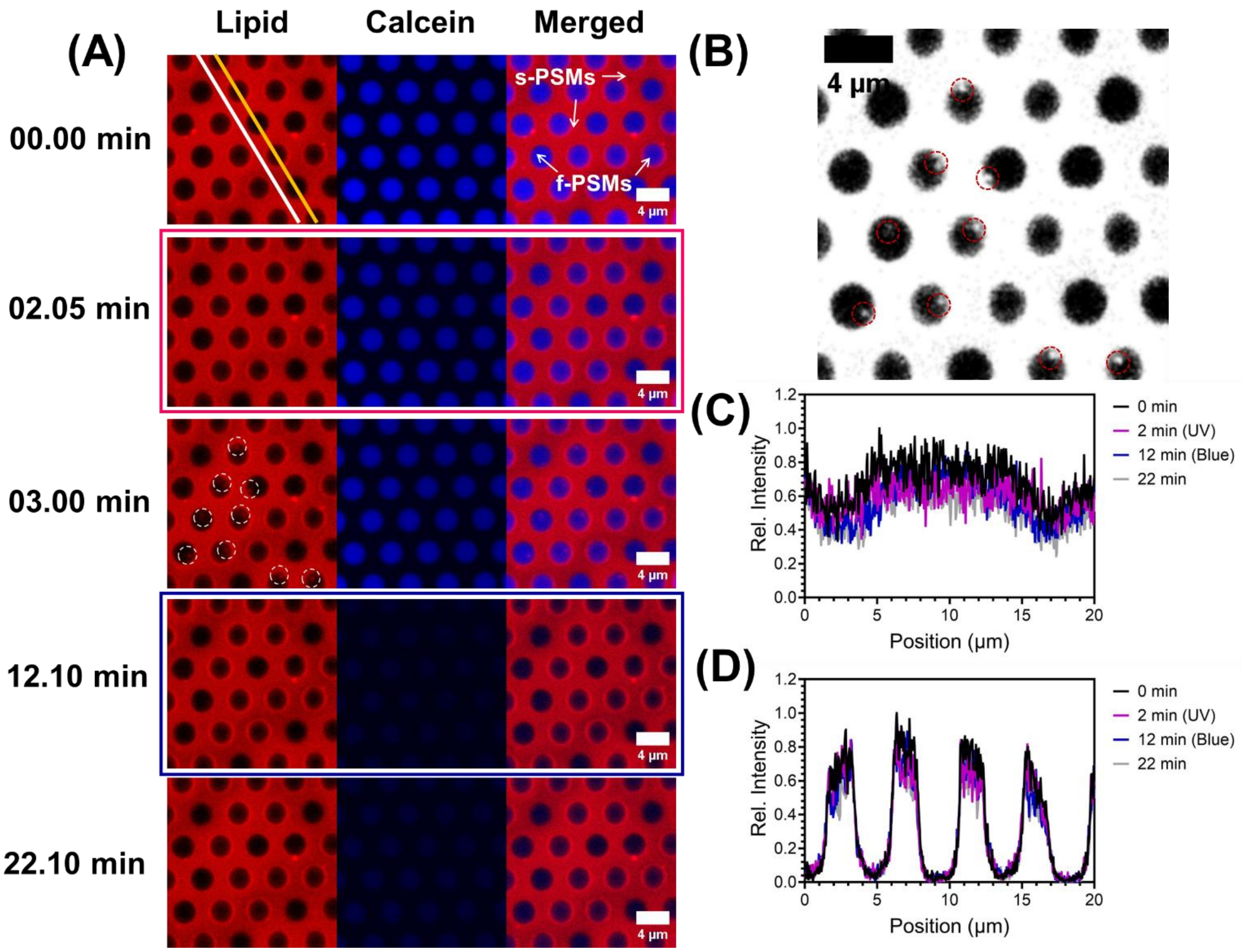

FIGURE S3 Irradiation of phase-separated PSMs in the presence of photo-Gb_3. Lipid composition: DOPC/SM_{18}/cholesterol/photo-Gb_3/ATTO655-DOPE (37:20:22:20:1, *n/n*). (A) Fluorescence images of PSMs (red fluorescence, lipid) and the corresponding calcein fluorescence (blue fluorescence, calcein) and the merged images before irradiation, following exposure to UV light (magenta), blue light (blue) and at the end of the experiment (Movie S2). A contrast correction factor (γ) of 0.5 was applied for better visualization. (B) After Gaussian blurring, a binary image of the lipid channel at 3 min was generated to properly detect l_d-lakes (dashed circles) after UV irradiation. (C) Evolution of the fluorescence intensity profile of s-PSMs (white line) and (D) f-PSMs (yellow line) throughout an irradiation cycle.

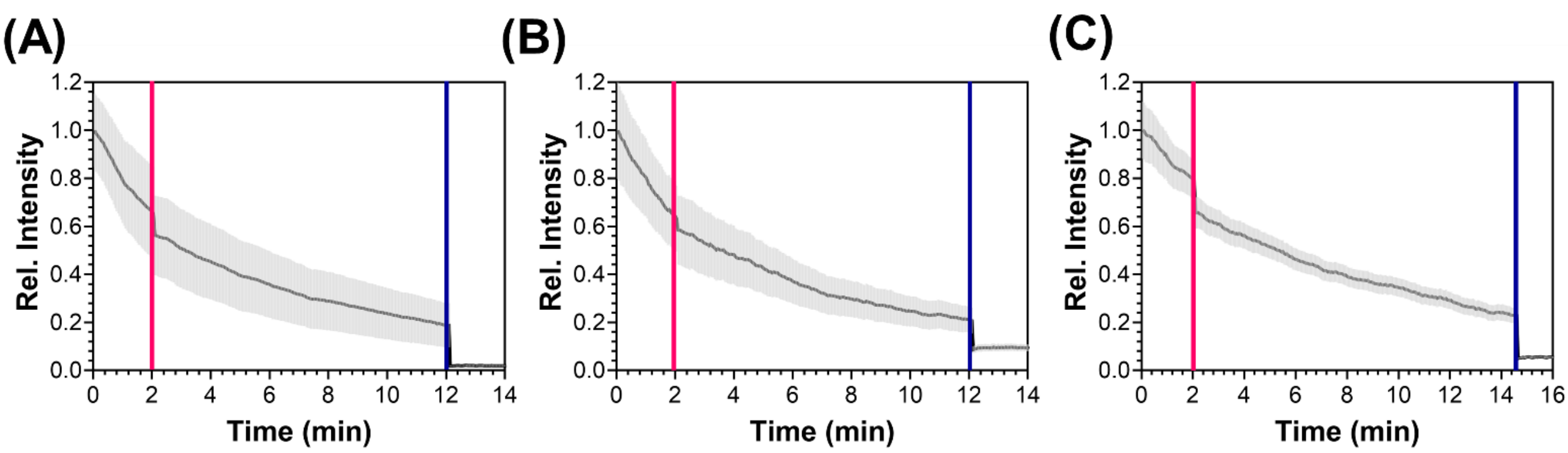

FIGURE S4 Influence of the illumination process on the calcein fluorescence in PSMs. The relative calcein intensity was reported as a function of time during imaging. (A) Control PSMs, *N = 20*. Composition: DOPC/SM_{18}/cholesterol/ATTO655-DOPE (39.5:39.5:20:1, *n/n*). (B) PSMs containing photo-Gb_3, *N = 16*. Composition: DOPC/SM_{18}/cholesterol/photo-Gb_3/ATTO655-DOPE (37:20:22:20:1, *n/n*). (C) Protein-enriched PSMs, *N = 19*. Composition: DOPC/SM_{18}/cholesterol/photo-Gb_3/ATTO655-DOPE (37:20:22:20:1, *n/n*), *c*(STxB) = 300 nM. The vertical lines mark the short irradiation with UV (magenta) and blue light.

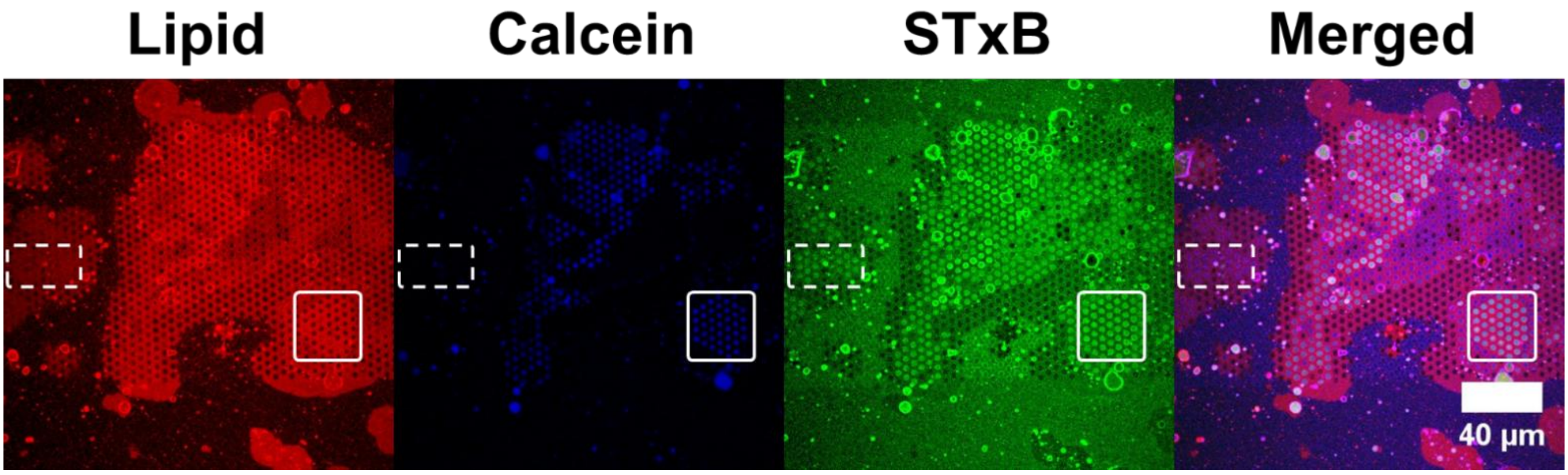

FIGURE S5 Selection of PSMs after STxB binding. Overview of a SiO_x-functionalized porous substrate after GUV sedimentation and incubation with STxB. Composition: DOPC/SM_{18}/cholesterol/photo-Gb_3/ATTO655-DOPE (37:20:22:20:1, *n*/*n*). *c*(STxB) = 300 nM. The entrapment of the water-soluble dye calcein indicates impermeable f-PSM areas (solid squares) that were chosen for further analysis. If calcein was not entrapped, even if the red and green fluorescence suggests the formation of membranes (dashed squares), these areas were discarded. A contrast correction factor (γ) of 0.5 was applied for better visualization.

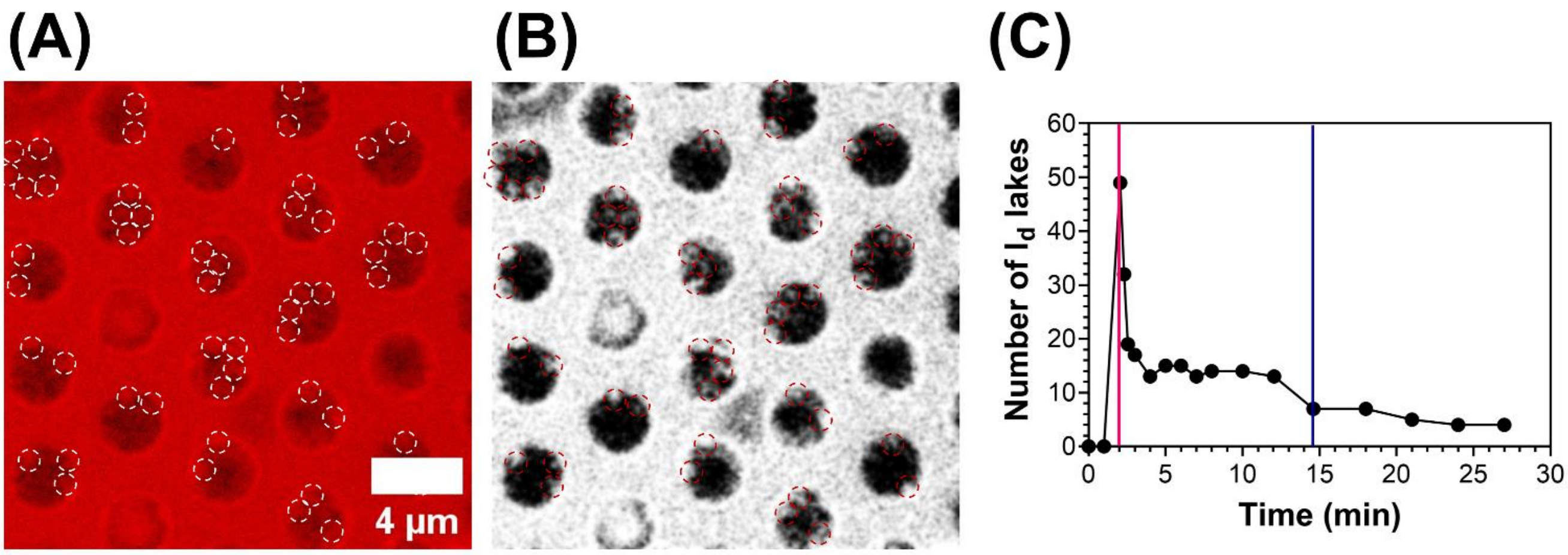

FIGURE S6 Isomerization of photo-Gb_3 in proteo-PSMs. Lipid composition: DOPC/SM_{18}/cholesterol/photo-Gb_3/ATTO655-DOPE (37:20:22:20:1, *n*/*n*). *c*(STxB) = 300 nM. (A) Transient l_d-lakes (white dashed circles) are visible in f-PSMs (dark) 30 s after *trans*-to-*cis* isomerization of photo-Gb_3. (B) For better visualization of the l_d-lakes (red dashed circles) and quantification, a greyscale image was generated with Gaussian blurring. (C) Quantification of l_d-lakes during the illumination process (*N* = 19 f-PSMs). The number of l_d-lakes increased immediately after UV irradiation (magenta line). The l_d-lakes persisted for a few seconds and about 70% of them have vanished after 10 min. A small portion (~10%) of l_d lakes persisted after blue light irradiation (blue line).

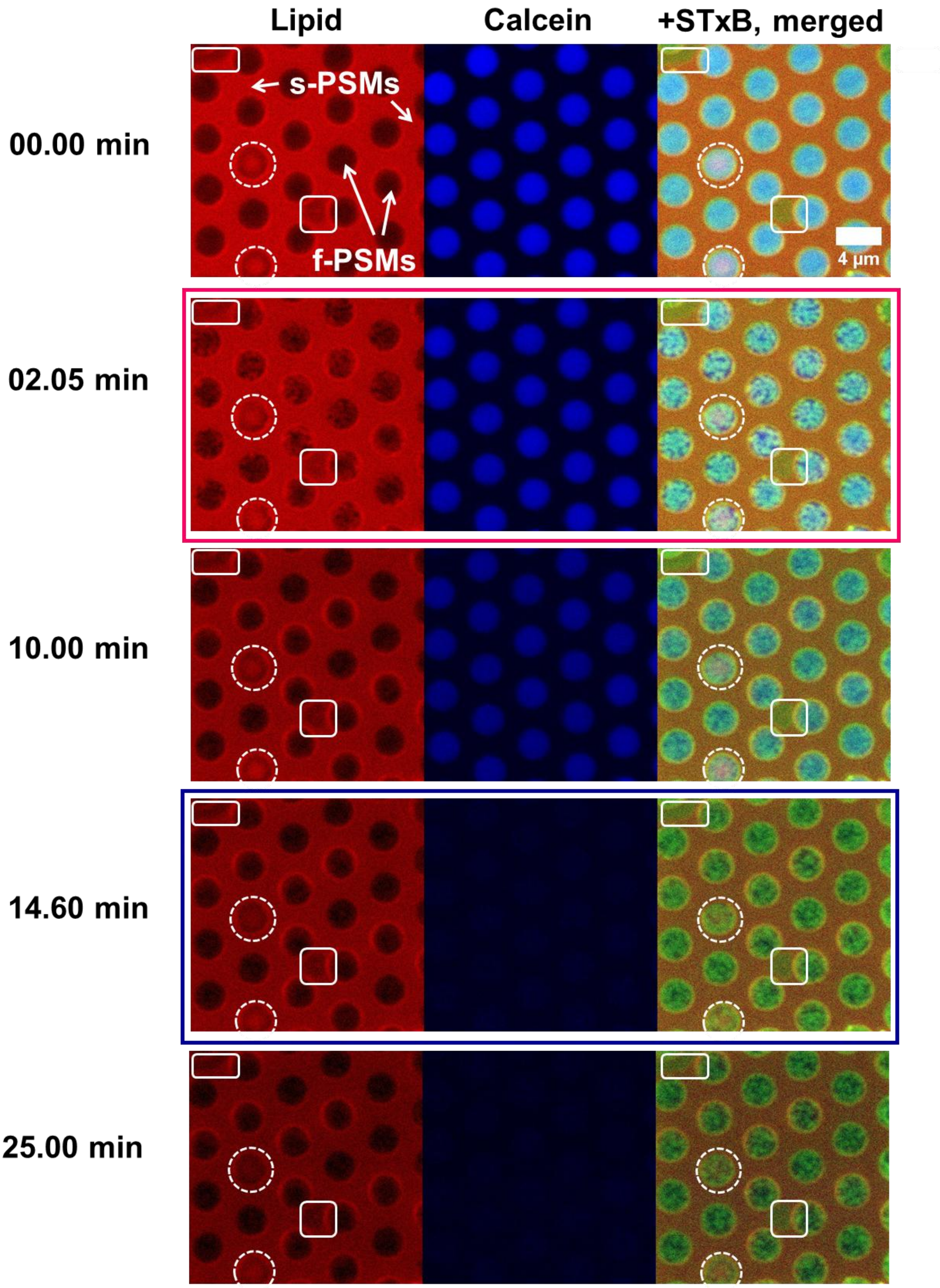

FIGURE S7 Irradiation of phase-separated PSMs with bound STxB. Lipid composition: DOPC/SM_{18}/cholesterol/photo-Gb_3/ATTO655-DOPE, 37:20:22:20:1, *n*/*n*. *c*(STxB) = 300 nM. The l_d-phase, labeled with ATTO655-DOPE (red fluorescence), is located on the s-PSMs, while f-PSMs are composed of the l_o-phase from which the fluorescent dye is excluded. Calcein: The water-soluble dye calcein (blue fluorescence) is entrapped in the micro cups. Merged: All fluorescence intensities are overlaid, including that of STxB (green), which bound to the l_o-phase of the f-PSMs. The white squares highlight a rarely observed ordered domain with bound STxB on an s-PSM. The white circles highlight the rarely observed bend f-PSMs that do not show the characteristic l_o-phase indicated by the exclusion of the lipid dye. A contrast correction factor (γ) of 0.5 was applied for better visualization.
